# Unveiling Zn Incorporation in CuInS_2_ Quantum Dots: X‑ray and Optical Analysis of Doping Effects, Structural Modifications, and Surface Passivation

**DOI:** 10.1021/acs.chemmater.5c01878

**Published:** 2026-03-09

**Authors:** Andrés Burgos-Caminal, Brener R. C. Vale, André F. V. Fonseca, Juan F. Hidalgo, Elisa P. P. Collet, Lázaro García, Víctor Vega-Mayoral, Saül Garcia-Orrit, Iciar Arnay, Juan Cabanillas-González, Laura Simonelli, Ana Flávia Nogueira, Marco Antônio Schiavon, Thomas J. Penfold, Lazaro A. Padilha, Wojciech Gawelda

**Affiliations:** † Madrid Institute for Advanced Studies IMDEA Nanoscience, 202533 Ciudad Universitaria de Cantoblanco, Calle Faraday 9, 28049 Madrid, Spain; ‡ Departamento de Química, Universidad Autónoma de Madrid, Ciudad Universitaria de Cantoblanco, Calle Francisco Tomás y Valiente 7, 28049 Madrid, Spain; § Departamento de Química Analítica, Química Física e Ingeniería Química, Universidad de Alcalá, Alcalá de Henares, 28805 Madrid, Spain; ∥ Departamento de Química Física Aplicada, Universidad Autónoma de Madrid, Ciudad Universitaria de Cantoblanco, Calle Francisco Tomás y Valiente 7, 28049 Madrid, Spain; ⊥ Instituto de Física Gleb Wataghin, Universidade Estadual de Campinas—UNICAMP, Campinas 13083-852, São Paulo, Brazil; # Grupo de Pesquisa Química de Materiais, Departamento de Ciências Naturais, Universidade Federal de São João Del-Rei, São João Del-Rei 36301-160, Minas Gerais, Brazil; ∇ CELLS-ALBA Synchrotron Light Source, Cerdanyola del Vallès, 08290 Barcelona, Spain; ○ Laboratório de Nanotecnologia e Energia Solar, Chemistry Institute, University of Campinas—UNICAMP, Campinas 13083-970, São Paulo, Brazil; ◆ Chemistry, School of Natural and Environmental Sciences, 5994 Newcastle University, NE1 7RU Newcastle upon Tyne, U.K.; ¶ Faculty of Physics, Adam Mickiewicz University, ul. Uniwersytetu Poznańskiego 2, 61-614 Poznań, Poland

## Abstract

CuInS_2_ quantum dots (QDs) have gained significant attention owing to their remarkable broadband emission, making them desirable for various optoelectronic applications requiring efficient luminescent nanomaterials. However, maximizing radiative recombination in CuInS_2_ QDs necessitates minimizing intragap trap states. A common approach involves the introduction of Zn during the synthesis, which typically promotes the formation of a ZnS shell that passivates the QD surface. Despite its importance, the characterization and quantification of Zn incorporation using conventional techniques, such as optical spectroscopy or electron microscopy, remains challenging. In this study, we utilized X-ray absorption spectroscopy, in both X-ray absorption near-edge structure and extended X-ray absorption fine structure spectral ranges, to investigate Zn incorporation into CuInS_2_ QDs, probing at the Zn, S, and Cu K-edges. This approach allowed us to detect the formation of a ZnS surface shell, tentatively quantifying its thickness, and to distinguish between Zn as a substituent at the shell or as an interstitial defect. Additionally, we explored the dynamical properties of CuInS_2_ QDs using time-resolved optical spectroscopies, particularly in the presence of electron and hole acceptors (benzoquinone and phenothiazine), observing that hole transfer is highly sensitive to shell thickness. These results provide deeper insights into the Zn-induced shell.

## Graphical abstract

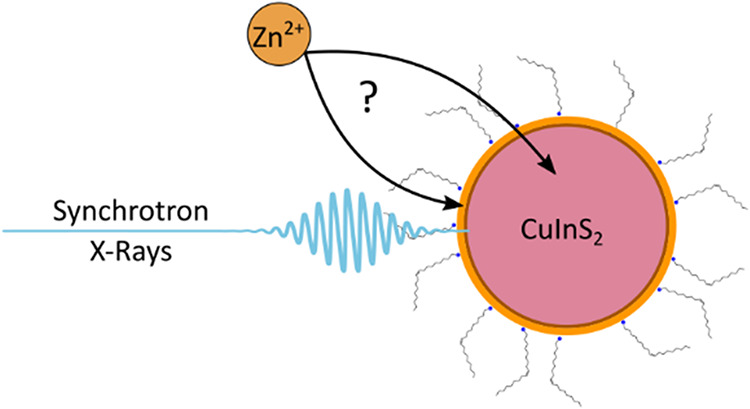

## Introduction

CuInS_2_ quantum dots (CIS QDs) have been intensively studied for different technological applications,^1^ such as light-emitting diodes,^2,3^ luminescent solar concentrators,^4–6^ QD-sensitized solar cells,^7,8^ fluorescent probes,^9,10^ nanothermometers,^11^ or cell imaging.^12–14^ One of the most important aspects in many of these applications, especially in optoelectronics, is the photoluminescence (PL) quantum yield, which is given by the ratio between emitted and absorbed photons.^15^ Photons are generally emitted through the radiative recombination of excitons inside the QD. Any other process that involves exciton recombination without the emission of a photon will be detrimental and decrease the PL quantum yield (PLQY). Typically, at low excitation fluences, nonradiative recombination is dominated by trap-assisted recombination akin to the Shockley-Read-Hall mechanism in bulk semiconductors.^16^ Although the defect states involved in this mechanism can be present inside the core of the QD, they are most often localized on the surface, due to the unbound orbitals of the surface atoms. A common strategy to passivate surface defects is to employ a shell of a larger bandgap semiconductor material surrounding the core of the QD, to prevent the formation of surface trap states, and generally confine the photoexcited charge carriers inside the core.^17,18^

CIS QDs are typically passivated with a ZnS shell, although other structures such as CuInS_2_/CdS, have also been reported.^19^ ZnS is particularly favorable due to the similar structure of ZnS zinc blende and CIS chalcopyrite. A progressive blue shift of the emission with the formation of the shell has been interpreted as ZnS forming an alloy with a composition gradient across the interface, instead of a clear and distinct shell.^20^ Indeed, according to Berends et al.,^21^ the reaction growth of ZnS with CIS QDs is more complex than that of other QDs, and this reaction can result in alloy formation, cation exchange, etching, and core–shell formation. Furthermore, Zn^2+^ cations are often considered to replace Cu^+^ and In^3+^. This can occur when applying a post-treatment with a Zn^2+^ precursor on previously synthesized CIS QDs, which gradually forms the ZnS shell.^20,22,23^

Usually, these different surface reactions are followed by: (a) indirect measurements such as UV–Vis absorption, and emission spectroscopies, or (b) more direct and structure-sensitive techniques such as X-ray diffraction, X-ray photoelectron spectroscopy, and transmission electron microscopy (TEM). However, these latter methods can only be used with solid samples, whereas optical probes can also be used in colloidal dispersion. Measuring physicochemical properties in colloidal suspension is advantageous because the properties of QDs in the solid state may be affected by the different dielectric environment.^24^ Despite their inherent limitations, PL and UV–Vis absorption spectroscopy remain the most widely utilized techniques for probing the optical properties and electronic transitions in QDs. However, the availability of more advanced and selective characterization methods—particularly those capable of analyzing nanocrystals within colloidal dispersions—is critically important for gaining deeper insights into their structural, compositional, and dynamic properties. Here, we employed steady-state X-ray absorption spectroscopy (XAS) to investigate colloidal CIS QDs. We exploit several unique advantages of XAS, such as elemental specificity, sensitivity to oxidation states, and local atomic structure, compared to UV–Vis spectroscopy.^25^

To maximize the obtained information, we probed both the X-ray absorption near-edge structure (XANES) and the extended X-ray absorption fine structure (EXAFS) spectral regions.^25^

We have studied five different samples, by varying their stoichiometries and Zn-doping levels, to systematically characterize and corroborate the optical properties of the material. Our main results deliver new insights into the role and incorporation of Zn atoms into CIS QD structures. We observed them both at the surface and inside the core, depending on the Cu:In stoichiometry. We probed these effects using complementary steady-state UV–Vis and X-ray spectra as relevant observables. Finally, we correlated these structural and compositional changes in CIS QDs with the charge transfer efficiency toward electron and hole acceptors using time-resolved and steady-state optical spectroscopies.

## Experimental Section

The following methods and setups have been published previously.^26,27^ The descriptions included below were taken from these two articles.

### CIS/Core Synthesis

First, 0.0587 g of InCl_3_ and 0.0238 g (1:1 Cu:In; 100%) or 0.004 g (0.2:1 Cu:In; 20%) of CuCl were weighed and placed in a 50 mL 3-neck flask. To this flask, 8 mL of 1-octadecene (ODE), 60 μL of oleic acid (OA), and 250 μL of dodecanethiol (DDT) were also added. The mixture was dried under vacuum at 90 °C for 30 min. During these 30 min, a mixture with 0.038 g of sulfur (S) in 3 mL of oleylamine (OAm) was subjected to ultrasound for 5 min. After this time, the solution of precursors in ODE was heated to 180 °C under an argon atmosphere for 5 min. Then, the temperature was lowered to 160 °C and 2 mL of the S-OAm solution were injected into the flask, monitoring for 10 min. The solution was cooled in an ice bath to room temperature (25 °C), under stirring and in an inert atmosphere (Argon).

After the synthesis step, the suspension was transferred to a Falcon centrifuge tube and 8.0 mL of isopropanol were added to purify the NCs. The tube was then taken to the centrifuge for 10 min at 7000 rpm. Finally, the supernatant was removed, and the nanoparticles were suspended in cyclohexane.

### CZIS/Core Synthesis

First, 0.0587 g of InCl_3_, 0.0238 g (1:1 Cu:In; 100%) or 0.004 g (0.2:1 Cu:In; 20%) of CuCl, and 0.0274 g of ZnCl_2_ were weighed and placed in a 50 mL 3-neck flask. To this flask, 8 mL of 1-octadecene (ODE), 60 μL of oleic acid (OA), and 250 μL of dodecanethiol (DDT) were also added. The mixture was dried under vacuum at 90 °C for 30 min. During these 30 min, a mixture of 0.0257 g of sulfur (S) in 2 mL of oleylamine (OAm) was subjected to ultrasound for 5 min. After this time, the solution in the 3-neck flask was heated to 180 °C under an argon atmosphere for 5 min. Then, the temperature was adjusted to 160 °C, and 2 mL of the S-OAm solution was injected into the flask, allowing it to react for 10 min. The solution was cooled in an ice bath to room temperature (25 °C), under stirring and in an inert atmosphere (Argon).

After the synthesis step, the suspension was transferred to a Falcon centrifuge tube and 8.0 mL of isopropanol were added to purify the NCs. The tube was then taken to the centrifuge for 10 min at 7000 rpm. Finally, the supernatant was removed, and the nanoparticles were suspended in cyclohexane.

### CZIS Core–Shell Synthesis

#### Synthesis of Zn-OAm Stock Solution

First, 0.2725 g of ZnCl_2_ were weighed and placed in a 50 mL 3-neck flask. To this flask, 4 mL of octadecene (ODE) and 1 mL of oleylamine (OAm) were also added. The mixture was dried under vacuum at 90 °C for 30 min. After this time, the solution was heated at 150 °C under an argon atmosphere for 10 min. Then, the temperature was adjusted to 50 °C.

### CZIS/ZnS Core–Shell Synthesis

The same procedure described above for the CZIS (item 2) was done, except for the purification step. With the pristine solution at room temperature and the Zn-OAm stock solution (item 3.1) at 50 °C, 5 mL of Zn-OAm solution were injected. Then, the system was heated to 200 °C and allowed to react for 30 min. Then, the solution was cooled to room temperature (25 °C) in an ice bath, under stirring in an inert atmosphere.

After that, Isopropanol was added in a 1:1 ratio to the nanocrystal suspension and centrifuged at 9000 rpm for 10 min. The supernatant was discarded, and the tube remained open for ∼5 min to dry isopropanol residues. The precipitate was suspended in cyclohexane.

### Transient Absorption Spectroscopy

Transient absorption measurements were conducted using a Clark-MXR CPA-1 regenerative amplifier. The fundamental of the laser (775 nm, 1 kHz, 120 fs, 1 mJ) was divided into two paths. One beam supplied a noncolinear optical parametric amplifier (NOPA) to generate 520 nm pulses, and the result was filtered to the desired fluence to pump the sample. The second beam was sent through a CaF_2_ crystal to generate a broadband supercontinuum by self-phase modulation spanning between 380 and 720 nm, which was used as the probe. Due to technical circumstances, the Cu_0.3_InS_2_ sample had to be probed with a supercontinuum generated with a sapphire crystal, limiting its bandwidth to 480–700 nm. A delay line was used to control the temporal delay between both pulses, which spatially overlapped on the sample. The probe pulse was divided before the sample position between a reference and a signal beam. The latter is sent through the sample, and both are collected into a prism spectrometer (Entwicklungsburo Stresing GmbH) with a double CCD array. A homemade software recorded the normalized change in absorption (Δ*A*) in a shot-to-shot configuration. All measurements were performed at magic angle (54.7°) between the pump and probe to avoid anisotropy effects. The samples were measured in 2 mm-thick quartz cuvettes with constant stirring with a magnetic bar perpendicular to the incident beam.

### Steady-State X-ray Absorption Spectroscopy

The steady-state XAS spectra were obtained at the BL-22 CLÆSS Beamline from the ALBA synchrotron in Barcelona (Spain).^28^ The X-ray beam is obtained from a multipole wiggler, and monochromatized with a double-crystal monochromator employing Si(111) crystals. The beam is focused down to a spot of 200 × 50 μm^2^ at the sample position. The samples are contained in closed liquid cells with Kapton windows, and the absorption is measured in total fluorescent yield mode. Three edges where measured during the same experiment: (1) Zn K-edge (9552–11036 eV), (2) Cu K-edge (8868 – 9640 eV), and (3) S K-edge (2435–2820 eV).

### Time-Resolved Photoluminescence

TRPL decay dynamics were obtained through time-correlated single photon counting (TCSPC) using a TimeHarp Picoquant multichannel time correlator. A 355 nm Nd:YAG pulsed laser from Teem Photonics was used as excitation at 1 kHz. PL detection was carried out at a single wavelength using a thermoelectrically cooled Hamamatsu photomultiplier coupled to a 0.5 m spectrometer (SP2500 Princeton Instruments, Acton Research) equipped with a 600 lines mm^–1^ grating.

### Steady-State Absorption and Photoluminescence

UV–Vis absorption spectra were recorded using a Varian Cary 5000 UV–Vis-NIR spectrophotometer (Agilent), while the photoluminescence spectra were recorded with a Hamamatsu PLQY spectrometer model C13534–11. All spectra were acquired at room temperature and in ambient conditions.

## Results and Discussion

In order to compare the photophysical properties of different synthesis stoichiometries and Zn doping, we prepared five different samples (Figure 1A): (A) stoichiometric CuInS_2_, (B) Cu-deficient CuInS_2_, (C) Zn-doped stoichiometric CuInS_2_, (D) Zn-doped Cu-deficient CuInS_2_, and (E) Zn-doped Cu-deficient CuInS_2_ with a post-treatment of ZnCl_2_ at 200 °C. This last post-treatment is expected to further passivate the surface and thus prevent nonradiative recombination and charge transfer to surface acceptors.^23^ The detailed synthesis method, which is based on previously published work, can be found in the Experimental section. Elemental analysis reveals that the synthesis under stoichiometric conditions produces Cu-rich samples (Table S1). However, Zn-incorporation results in a Cu:In stoichiometry closer to the nominal value from the synthesis. Considering these results, we have named the samples accordingly: (A) CuIn_0.4_S_2_, (B) Cu_0.3_InS_2_, (C) Cu­(Zn)­In_0.8_S_2_, (D) Cu_0.3_(Zn)­InS_2_, and (E) Cu_0.2_(Zn)­InS_2_/ZnS. The stoichiometry of Zn is not included in the formula due to the uncertainty of its incorporation at this point. High-resolution transmission electron microscopy (HR-TEM) measurements show sizes ranging from 3.3 ± 0.5 nm for Cu_0.3_(Zn)­InS_2_ to 5.3 ± 0.6 nm for CuIn_0.4_S_2_ (Figure S1). XRD results (Figure S2) show a qualitative agreement with a broadened chalcopyrite structure for the Cu-deficient samples. However, for Cu-rich samples, we observe an additional peak at 2θ ≅ 50°, which we attribute to a partial contribution of a wurtzite structure. A summary of synthetic conditions and characteristics is shown in Table 1.

**Figure 1:**
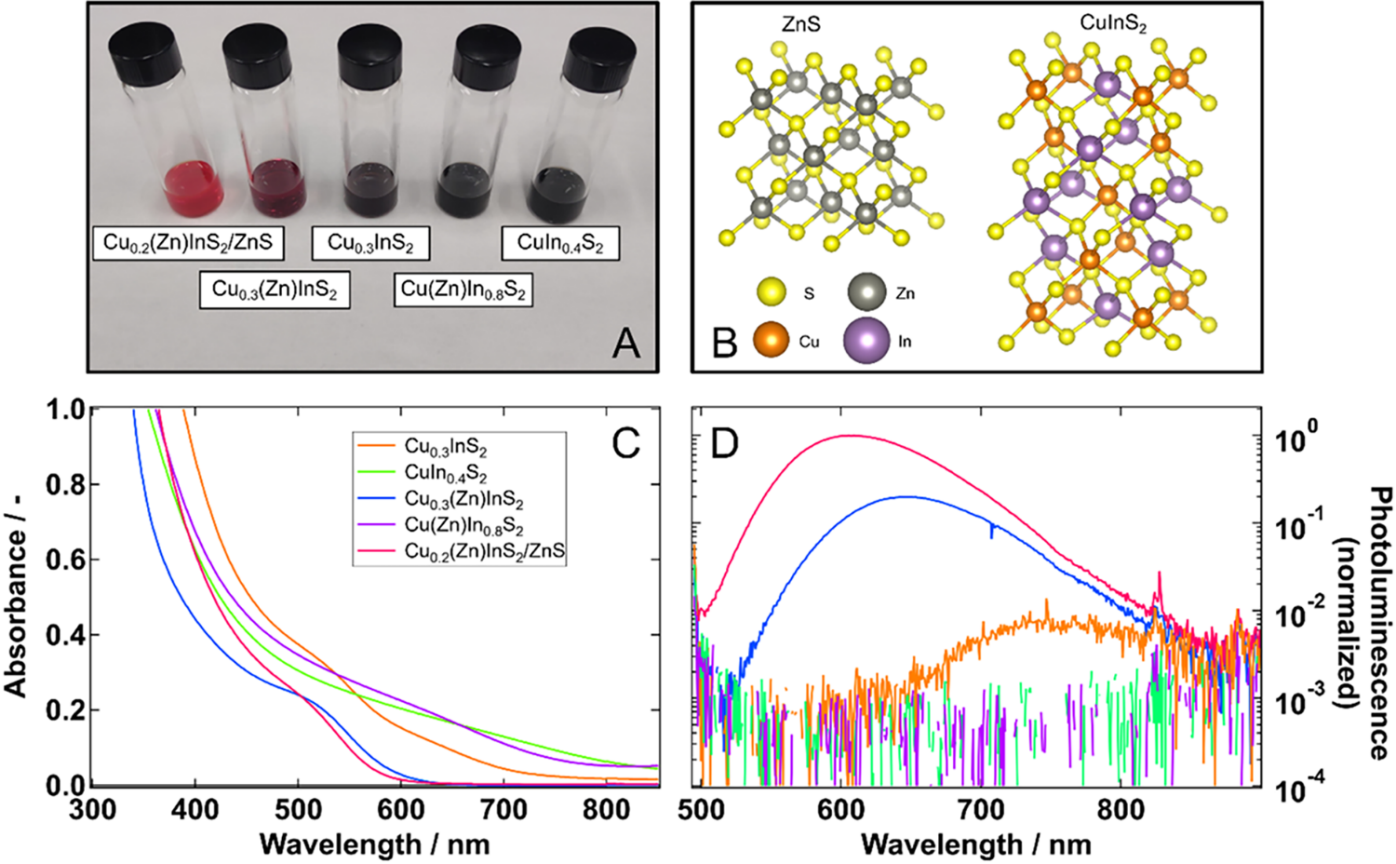
(A) Photograph of the five samples under study, and (B) expected crystalline structures of the CuInS_2_ core (chalcopyrite) and the ZnS shell (zincblende).^30–32^ The bottom panels show the steady state characterization of the CuInS_2_ samples Cu_0.2_(Zn)­InS_2_/ZnS (red), Cu_0.3_(Zn)­InS_2_ (blue), Cu­(Zn)­In_0.8_S_2_ (purple), Cu_0.3_InS_2_ (orange), and CuIn_0.4_S_2_ (green). (C) UV–Vis absorbance, and (D) log scale PL spectra of the studied samples with different levels of passivation. Note the correlation between the disappearance of the sub-bandgap tail in the absorption spectra (C) and the increase in the PL signals (D). The linear version of panel (D) is shown in Figure S4A.

**Table 1:** Summary of Synthetic Conditions and General Characteristics^a^

QD	Cu:In synthetic stoichiometry	Cu:In measured stoichiometry	dandgap (eV)	Zn-doping	Zn post-treatment	LSPR	size (nm)
CuIn_0.4_S_2_	1	2.41 ± 0.03	1.80	no	no	yes	5.32 ± 0.04
Cu_0.3_InS_2_	0.2	0.28 ± 0.03	1.97	no	no	no	3.72 ± 0.08
Cu(Zn)In_0.8_S_2_	1	1.25 ± 0.05	1.86	yes	no	yes	4.20 ± 0.06
Cu_0.3_(Zn)InS_2_	0.2	0.299 ± 0.009	2.19	yes	no	no	3.31 ± 0.04
Cu_0.2_(Zn)InS_2_/ZnS	0.2	0.245 ± 0.005	2.23	yes	yes	no	3.32 ± 0.03

a The bandgap is estimated from transient absorption spectroscopy measurements shown in Figure S3.

Figure 1 also shows the reported crystalline structures of the CuInS_2_ core and the ZnS shell (Figure 1B), as well as the UV–Vis absorption (Figure 1C) and PL (Figure 1D) spectra of the investigated samples. The absorption spectra show that Cu-deficient samples have sharper features and are blue-shifted compared to the Cu-rich ones. Zn-doped samples show further blue-shifted spectra compared to the ones without Zn. According to previous studies, this behavior is quite common for CIS QDs and is due to cation-exchange processes, in which Cu^+^ and In^3+^ are replaced by Zn^2+^ in the lattice structure.^22,23^ Density of state calculations have demonstrated that, for CIS QDs, Cu^+^ and S^2–^ contribute to the valence band (VB) edge, whereas In^3+^ dominates the conduction band (CB) edge, with a small contribution of Cu^+^ and S^2–^.^29^ However, once Zn^2+^ ions are present inside the lattice, forming an alloy, Zn^2+^ (3d) orbitals start to contribute to the CB-edge, which shifts it to higher energies with the increasing concentration of Zn^2+^ in the lattice.^29^ Lastly, only the Cu_0.2_(Zn)­InS_2_/ZnS and Cu_0.3_(Zn)­InS_2_ samples show significant photoluminescent properties among all the samples. By looking closer at the PL signals of all the samples in the log scale (Figure 1D), we can observe that the weak PL band of Cu_0.3_InS_2_ is slightly higher than the nondetectable ones for CuIn_0.4_S_2_ or Cu­(Zn)­In_0.8_S_2_. This indicates that both Cu deficiency and Zn doping are beneficial for reducing charge carrier trapping.

Furthermore, the steady state characterization in Figure 1 shows that a considerable PL signal appears only with both Zn doping and Cu deficiency. This also eliminates most of the considerable sub-bandgap absorption tails,^33^ which are assigned to defects and disorder in the CIS QDs. We experimentally obtained PLQY values of 30%, 7.4%, and 0.4% for Cu_0.2_(Zn)­InS_2_/ZnS, Cu_0.3_(Zn)­InS_2_, and Cu_0.3_InS_2_, respectively, in line with the passivation level and the PL spectra. On the other hand, both Cu-rich samples exhibit, what appears to be a localized surface plasmon resonance (LSPR) in the near-infrared (NIR), as shown in Figure S4B. Although somewhat unexpected, the occurrence of LSPR has been reported for samples with both Cu-deficiency, which is reported to lead to hole doping and eventually to the formation of surface plasmons,^34^ and In deficiency.^35^ Our results suggest it could likewise be caused by the excess of Cu in CIS QDs, providing electron doping. Furthermore, as reported by Wang and Swihart, such In deficiency tend to lead to CIS QDs with a Wurtzite structure, like the ones reported here.^35^

### Structural Investigation

In order to shed more light on the Zn doping effect and the details of its spatial distribution within the crystal lattices of the different QD structures, we carried out a systematic structural analysis, performing XAS experiments at the CLÆSS beamline of the ALBA synchrotron (Barcelona, Spain).^28^ Our aim was to correlate XAS signals originating from different atomic constituents of the QDs, i.e., from Zn, Cu, and S atoms, which can be found both inside and on the surface of the nanocrystals. Given the very broad energy range of CLÆSS (2.4–63 keV) and its versatile equipment for measuring XAS signals for both solid and liquid samples, we were able to record XANES and EXAFS spectra for colloidal dispersions of CIS QDs at Zn, S, and Cu K-edges in a single experiment. The most relevant for this study are the XAS signals for the Zn and S atoms (Cu is not shown here, except in Figure S5). The results for the Zn K-edge are shown in Figure 2, where Figure 2A depicts the normalized XANES spectra of the different Cu­(Zn)­InS_2_ (CZIS) samples studied and the corresponding signal for a ZnS reference. Figure 2B shows the Fourier transform of the corresponding *k* ^2^-weighted EXAFS oscillations.

**Figure 2:**
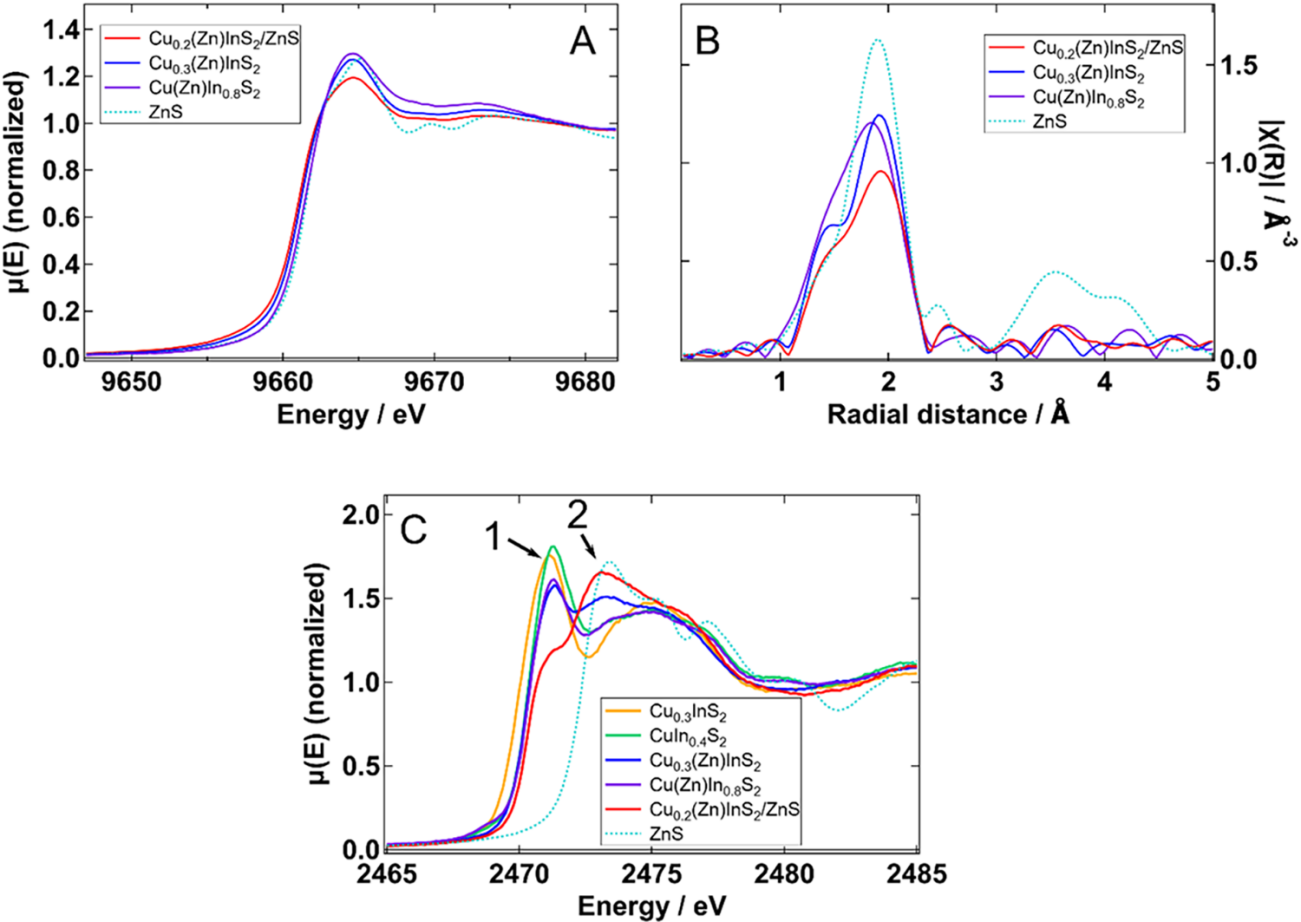
(A) Normalized XANES spectra at the Zn K-edge of the Zn-containing samples and bulk ZnS, and (B) the Fourier transforms of the corresponding *k* ^2^-weighted EXAFS oscillations. (C) Normalized XANES spectra at the S K-edge. Two arrows point to the characteristic bands originating from S atoms inside the QD (**1**) and within the ZnS shell on the surface (**2**).

For all CZIS QDs, the Fourier-transformed EXAFS signals contain not only the main peak, which originates from the scattering of the nearby Satoms (first coordination shell at around 1.9 Å), but also a non-negligible and reproducible shoulder at lower distances. This can be assigned to either impurities that bond to Zn with shorter bond lengths,^36^ or to Zn at interstitial or displaced positions.^37,38^ Although interstitial contributions would be expected to be detected at around 1 Å, it is hard to univocally identify them because of a combination of noise level and background subtraction, which can induce artifacts. Instead, the visualization of the corresponding wavelet transform^39^ can allow us to access finer details (see Figure 3, further below in the text).

**Figure 3:**
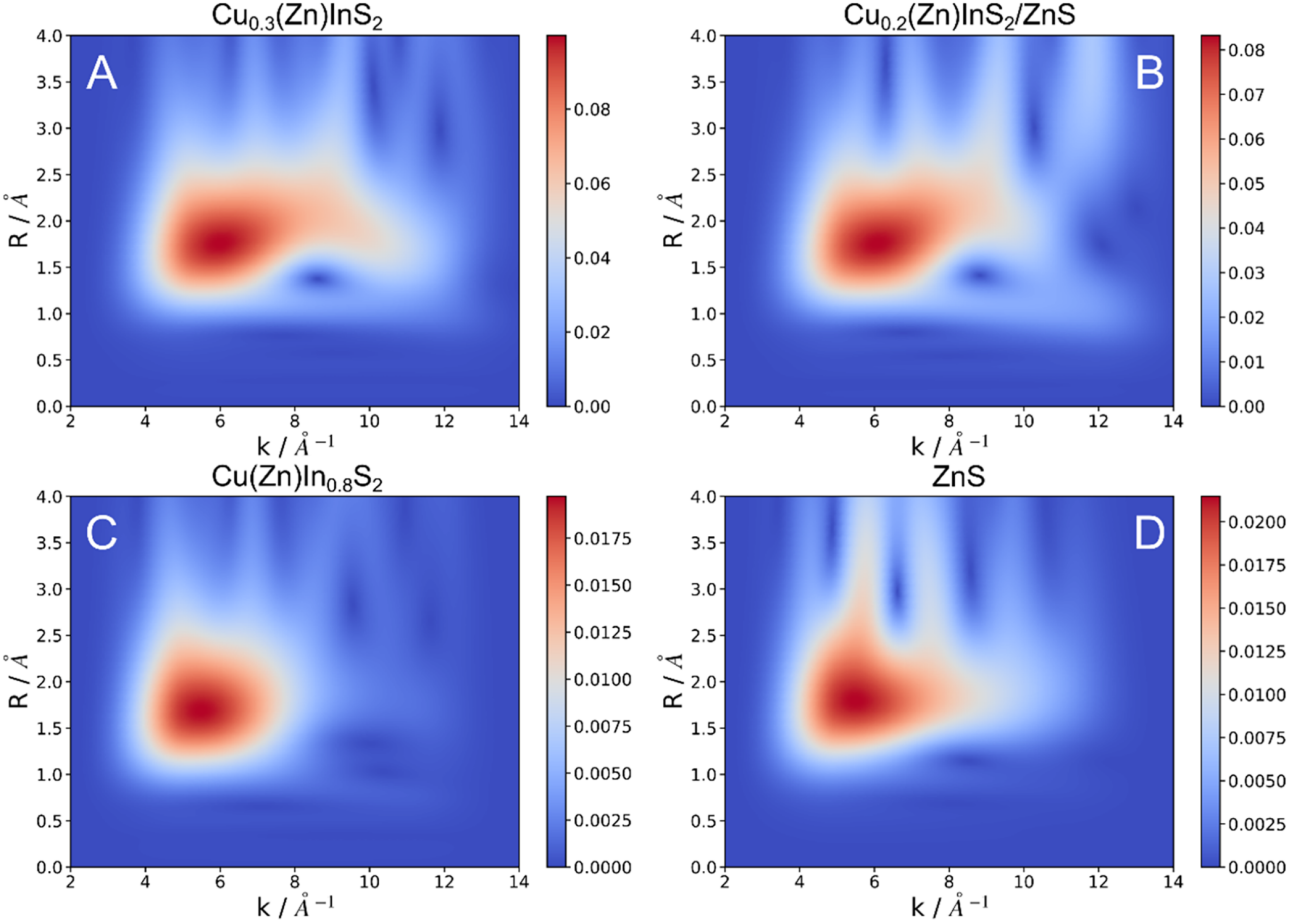
Wavelet transform of the Zn K-edge EXAFS of Cu_0.3_(Zn)­InS_2_ (A), Cu_0.2_(Zn)­InS_2_/ZnS (B), Cu­(Zn)­In_0.8_S_2_ (C), and ZnS (D).

Interestingly, comparing the three Zn-doped QDs, a larger difference between the Cu-rich (Cu­(Zn)­In_0.8_S_2_) and the Cu-deficient samples (Cu_0.3_(Zn)­InS_2_ and Cu_0.2_(Zn)­InS_2_/ZnS) can be observed. For the Cu-rich sample, the main peak shifts to lower distances and presents a more pronounced low-distance shoulder at the same time. This points to a different incorporation of Zn for the two cases. While for Cu­(Zn)­In_0.8_S_2_, Zn atoms are incorporated mainly inside the QDs, the other two cases (Cu_0.3_(Zn)­InS_2_ and Cu_0.2_(Zn)­InS_2_/ZnS) have a large proportion of Zn atoms at the surface, probably forming a shell of ZnS.

Qualitatively, this interpretation is further corroborated by comparing the positions of the main Fourier transform features for the Cu-deficient QDs and the ZnS bulk sample (Figure 2B). In order to access finer details, we have performed EXAFS fitting using FEFF simulations carried out with the Demeter package.^40^ In our model, we used a zincblende crystal structure of ZnS^32^ for fitting the first coordination shell only (Figure S6 and Table S2). The main output extracted from this analysis is the coordination number, which is ideally equal to 4. For the Cu­(Zn)­In_0.8_S_2_ sample, we obtained a fit result close to 4; however, for Cu_0.3_(Zn)­InS_2_, this number decreased to 3.5, and for Cu_0.2_(Zn)­InS_2_/ZnS, it decreased even further down to 2.6. This decrease in coordination number indicates an increased proportion of Zn at the QDs’ surface. The quality of our fits indicates that the actual structure surrounding Zn atoms is much more complex than the bulk structures used for the fit. Nonetheless, they can serve for a semiquantitative analysis of changes in the local coordination of Zn atoms.

Looking at the XANES spectra (Figure 2A), the three QD samples present a peak centered at 9664.5 eV. It corresponds to the dipole-allowed 1s → 4p transition and its position exhibits energy shifts, which can be correlated with the coordination.^41^ We observe that it broadens following Cu­(Zn)­In_0.8_S_2_ < Cu_0.3_(Zn)­InS_2_ < Cu_0.2_(Zn)­InS_2_/ZnS. This agrees with what is observed in the EXAFS region, and it denotes that as we increase the passivation, the fraction of Zn atoms present on the QDs’ surface increases. Contrary to Zn atoms localized inside the core, those on the surface are characterized by higher disorder and lower coordination numbers (see EXAFS results mentioned earlier).

We now turn our attention to S atoms and discuss the K-edge XANES spectra shown in Figure 2C. For all studied samples, we observe a main peak at 2471.3 eV (peak 1), which corresponds to the 1s → 3p dipolar transition.^42,43^ Filling the 3p orbitals of Sanions by ionic bonding decreases the intensity of this peak. For instance, S with a formal charge of −2 should exhibit a very low-intensity peak. Figure 2C shows that peak 1 decreases in the following order: CuIn_0.4_S_2_ > Cu_0.3_InS_2_ > Cu­(Zn)­In_0.8_S_2_ > Cu_0.3_(Zn)­InS_2_ > Cu_0.2_(Zn)­InS_2_/ZnS.

These observations can be explained in light of similar findings in other semiconductors, such as Cu_*x*_In_*y*_Se_2_ (CISe), with a chalcopyrite crystal structure, which behaves similarly to CIS.^42,44^ Yamazoe et al. showed that Se K-edge peak 1 intensities are related to the relative amounts of Cuand In in CuInSe_2_ structures and the Se coordination environment. CISe with a 3-fold coordinated Se and more Cu vacancies shows a higher intensity of peak 1 than that in CISe with a 4-fold coordinated Se and fewer Cu vacancies.^44^ Because S behaves in a similar way to Se, we can interpret the increase in intensity of peak 1 in CuIn_0.4_S_2_ and Cu­(Zn)­In_0.8_S_2_, as compared to Cu_0.3_InS_2_ and Cu_0.3_(Zn)­InS_2_, as an indication of a more distorted tetrahedral S environment for the Cu-rich samples. This conclusion is in line with what has been observed for the Cu K-edge in our previous publication.^26^ The origin of this peak could be a hybridization between the filled S 3p and the empty In 5s orbitals, introducing holes for the transition from the 1s of S. Such transition lies at the same energy and has the same shape in In_2_S_3_,^45^ although it is unclear why the intensity increases for the Cu-rich samples. Hybridizations between S^2–^ and Cu^2+^ lie at lower energies.^46^ In Figure S5. Yamazoe et al. also discuss the appearance of an additional peak,^44^ which in our case is present at 2475 eV. However, we do not include it in the discussion because it overlaps with other spectroscopic features. Alternatively, peak 2 (2473.1 eV), which is present and well-defined for samples Cu_0.3_(Zn)­InS_2_ and Cu_0.2_(Zn)­InS_2_/ZnS, corresponds to S in a ZnS environment,^43,47^ agreeing with the bulk ZnS spectrum. Therefore, this result corroborates that ZnS shells are formed at the QD surface for these two samples, with Cu_0.2_(Zn)­InS_2_/ZnS having a considerably larger thickness, also in agreement with the result of the Zn K-edge. This also provides direct evidence to explain their considerable PL signal strength due to increased surface passivation. Its presence in Cu­(Zn)­In_0.8_S_2_ is, however, very limited and difficult to confirm.

To further quantify the difference between Cu_0.3_(Zn)­InS_2_ and Cu_0.2_(Zn)­InS_2_/ZnS, a linear combination fit of the first one was carried out as shown in Figure S7. An acceptable fit was obtained with 46% of Cu_0.2_(Zn)­InS_2_/ZnS and 54% of CuIn_0.4_S_2_. This result reveals that, at most, Cu_0.3_(Zn)­InS_2_ has half the amount of S on a ZnS shell than Cu_0.2_(Zn)­InS_2_/ZnS. We also attribute the difficulty in obtaining high-quality fit results using either linear combination fitting of the other QDs or a reconstruction from principal component analysis to an imperfect shell formed in the case of Cu_0.3_(Zn)­InS_2_, as compared to Cu_0.2_(Zn)­InS_2_/ZnS, which corresponds to a fully passivated case. Nonetheless, this method stands as a more direct and reliable way to detect the presence of a surface shell layer and to study the thickness of the formed ZnS shell compared to TEM, where it is difficult to distinguish the layers, requiring a very high resolution and the combination with spectroscopy.^48^ Furthermore, we can extract an approximate spectrum of S in the ZnS environment by carrying out different subtractions, obtaining a good agreement with the reference (Figure S8). Using the results in Figures S7 and S8, we can obtain rough estimates of the shell thickness (Table S3), obtaining values of 0.44 and 0.19 nm for Cu_0.2_(Zn)­InS_2_/ZnS and Cu_0.3_(Zn)­InS_2_. Comparing these values to the unit cell size of 0.54 nm, we can deduce that Cu_0.2_(Zn)­InS_2_/ZnS will have a complete passivation of the surface, while for Cu_0.3_(Zn)­InS_2_, it is only partial.

So far, we have established that the Zn^2+^ cations introduced during the synthesis form ZnS shells with different thicknesses for both Cu_0.3_(Zn)­InS_2_ and Cu_0.2_(Zn)­InS_2_/ZnS. However, we can obtain further details on their incorporation by performing more advanced analysis of the different Zn K-edge EXAFS signals using the wavelet transform (WT)^39^ (Figure 3). A WT is similar to the Fourier transform usually carried out in EXAFS (Figure 2B), but yields a 2D result resolved at which photoelectron wavevector (*k*) specific radial distance (*R*) appear. This is achieved using wavelets that serve as kernels for the integral transformation, effectively windowing it to a certain *k* region. This transformation is then carried out varying the center *k* of the wavelet in order to obtain the maps shown in Figure 3.^49^ This procedure can help to highlight EXAFS features and distinguish between photoelectron scattering with different elements, as heavier elements scatter at higher values of *k*.^39,49–51^

This gives us further insight into the incorporation of Zn into the core of the particles. Both Cu_0.3_(Zn)­InS_2_ and Cu_0.2_(Zn)­InS_2_/ZnS exhibit the most complex spectra, which are reminiscent of a mixture of the map for Cu­(Zn)­In_0.8_S_2_ and that of ZnS. Once again, the similarity with ZnS shows the formation of a shell of this material in both QD samples. Furthermore, both Cu-deficient samples present a weak signal around *R* = 1 Å and *k* = 4–12 Å^–1^. When analyzing WT of EXAFS data, the value of *k* is related to the atomic mass of the scatterer.^50,51^ Thus, while most of the signal can be related to the scattering with the light S atoms (*k* = 5–7 Å^–1^), this one would partly come from scattering off much heavier atoms, such as Zn, Cu, or In, at shorter distances. Therefore, we propose it originates from Zn atoms incorporated as interstitial defects, in closer proximity to those other metal atoms. Note that the effective ionic radius of Zn^2+^ is 0.6 Å,^52^ making interstitial distances of 1 Å plausible. Interestingly, Cu­(Zn)­In_0.8_S_2_, shows an even weaker signal from these defects. In this case, Zn must be mainly incorporated as a substituent of Cu or In. Indeed, previous studies considered that Zn was being introduced as a substituent, eventually forming a ZnS shell, after sufficient substitution of Cu^+^ and In^3+^ ions.^20^ An alternative data treatment enhances these features in Figure S9. It is worth noting that the same effect is observed for the wavelet transforms of the Cu K-edge EXAFS data (Figure S10). Once again, the two samples that show an elongated signal around *R* = 1 Å and *k* = 10–12 Å^–1^ are Cu_0.3_(Zn)­InS_2_ and Cu_0.2_(Zn)­InS_2_/ZnS. Therefore, when looking at the photoelectrons emitted from Cu atoms, we can also observe the effect of the closely lying interstitial Zn defects. Thus, they are also introduced in the core of the QDs for these two samples and are not only present at the ZnS shell. Furthermore, these two samples, and to a lesser extent Cu_0.3_InS_2_, show a clear scattering at *k* = 10–12 Å^–1^ over larger distances, indicating a higher presence of other heavy atoms (Cu, In, Zn) at the typical Cu–S distance. The accumulation at the highest *k* values (*k* = 12 Å^–1^) compared to the weaker signal at *R* = 1 Å, and the absence at the Zn K-edge, may suggest that this signal stems from interstitial In^3+^ ions that approach the Cu position in Cu-deficient samples. This analysis underlines the importance of measuring multiedge XAS data, especially for composite materials such as QDs, which facilitates more advanced structural analysis by correlating signals originating from different atomic constituents of the particle, both in the core and on its surface.

### Implications of Structural Modifications on Charge Carrier Dynamics and Surface Passivation

In order to further assess the level and nature of surface passivation in the two different Zn-doped Cu-deficient samples used in our studies, we have complemented our static PL measurements with transient absorption spectroscopy (TAS) and time-resolved photoluminescence (TRPL) studies using additional electron (benzoquinone, BQ) and hole (phenothiazine, PTZ) acceptors. The former technique allowed us to observe the overall quenching of the PL signal, while the latter two allowed us to observe the decay kinetics of the photoexcited QDs on two different time scales: (i) on ultrafast (<1 ns) with TAS and (ii) on nanosecond (>1 ns) with TRPL. We carried out the PL measurements using the same PLQY spectrometer that was used to obtain the results shown in Figure 1 and, thus, to maximize the accuracy. In steady-state PL, we observe a complete quenching of the signals with the addition of BQ, and a moderate effect with PTZ, mainly on Cu_0.3_(Zn)­InS_2_ (top panels in Figure 4 and Table 2). In time-resolved data, we observe a fast and efficient, ∼97%, PL quenching by electron transfer using BQ for the Cu_0.2_(Zn)­InS_2_/ZnS sample. We can discard energy transfer because the absorption of BQ is of higher energy and does not overlap with the emission of CZIS QDs. The addition of BQ to the colloidal dispersion of Cu_0.3_(Zn)­InS_2_ produced a chemical degradation and agglomeration, preventing us from carrying out any electron transfer study in it. Thus, the shell in this sample is insufficient to prevent chemical attacks beyond electron transfer. The effect of PTZ in the TRPL measurements is small in all cases, producing a quenching of the integrated TRPL of 4% and 13%, for Cu_0.2_(Zn)­InS_2_/ZnS and Cu_0.3_(Zn)­InS_2_, respectively (Table 2). Slightly higher quenching efficiencies are obtained from TRPL lifetime fitting as seen in the SI (Section 7). Alternatively, since TAS is sensitive to the sum of the distribution function of electrons and holes, and the ratio of effective masses is *m*_h_/*m*_e_ = 8, TAS is not very sensitive to the dynamics of holes.^53,54^ This explains why we observe a much larger effect with BQ and Cu_0.2_(Zn)­InS_2_/ZnS, but only a small one is seen for Cu_0.3_(Zn)­InS_2_ with PTZ.

**Figure 4:**
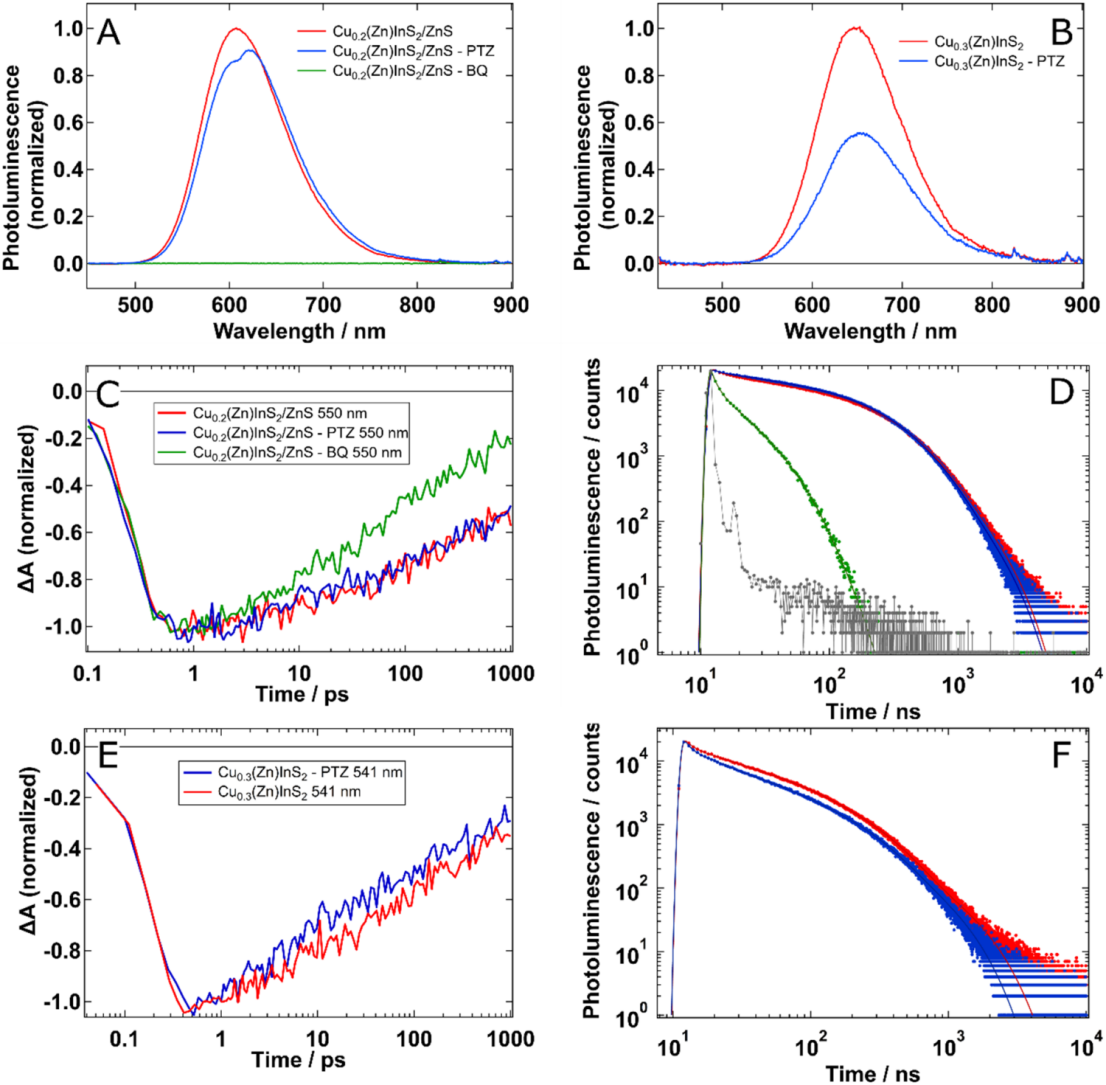
Top two panels: fluorescence quenching in Cu_0.2_(Zn)­InS_2_/ZnS (A) and Cu_0.3_(Zn)­InS_2_ (B). Bottom four panels: transient absorption (left, λ_pump_ = 520 nm, fluence = 80 μJ·cm^–2^) and time-resolved photoluminescence (right, λ_pump,_ = 355 nm) measurements for Cu_0.2_(Zn)­InS_2_/ZnS (B, C) and Cu_0.3_(Zn)­InS_2_ (E, F) with an electron acceptor (BQ, only for Cu_0.2_(Zn)­InS_2_/ZnS) and a hole acceptor (PTZ). The instrument response function (IRF) is shown in gray. While the effect of the electron acceptor is clear, the effect of the hole acceptor seems limited on the time-resolved measurements, slightly observed in Cu_0.3_(Zn)­InS_2_.

**Table 2:** Quenching by the Electron and Hole Acceptors BQ and PTZ^a^

QD	QD-PTZ	QD-BQ
PL	Φ_q_	Φ_q_
Cu_0.2_(Zn)InS_2_/ZnS	0.06	1
Cu_0.3_(Zn)InS_2_	0.4	
TRPL	Φ_q_	Φ_q_
Cu_0.2_(Zn)InS_2_/ZnS	0.04	0.97
Cu_0.3_(Zn)InS_2_	0.13	

a While the effect of BQ is large, extracting many electrons, the effect of PTZ is more limited for Cu_0.2_(Zn)­InS_2_/ZnS and further decreases in TRPL measurements due to hole localization. The PL results are the average of the three measured excitation wavelengths (420, 490, and 510 nm). The TRPL Φ_q_ is calculated from the integration of the TRPL traces. It considers equal emission at ca. 1 ns and thus evaluates the quenching after that. Alternatively, Φ_q_ calculated from lifetime fitting is shown in Table S4.

Taking into account the band alignment of CuInS_2_ and ZnS, shown in Scheme 1A, in addition to all the presented results, we can consider that (a) a shell of ZnS is being formed, which efficiently blocks hole and electron trapping at the surface in Cu_0.2_(Zn)­InS_2_/ZnS, and to a slightly lower degree in Cu_0.3_(Zn)­InS_2_; and (b) this does not prevent all charge transfer to suitable acceptors. Indeed, because we observe an efficient electron transfer in Cu_0.2_(Zn)­InS_2_/ZnS, electrons can, in principle, tunnel the ZnS barrier efficiently. Meanwhile, holes can also transfer in Cu_0.3_(Zn)­InS_2_ due to its small shell thickness, whereas this effect is marginal in Cu_0.2_(Zn)­InS_2_/ZnS. However, to understand the dramatic difference between electron and hole transfer dynamics, we need to consider the photophysical mechanisms in CIS QDs. According to the most accepted model, after excitation at the band edge, an exciton is created. Then, the hole is trapped into a confined hole state (CHS) related to a Cu defect, and the emission occurs from the electron delocalized in the conduction band and the hole in the CHS.^55,56^

**Scheme 1:**
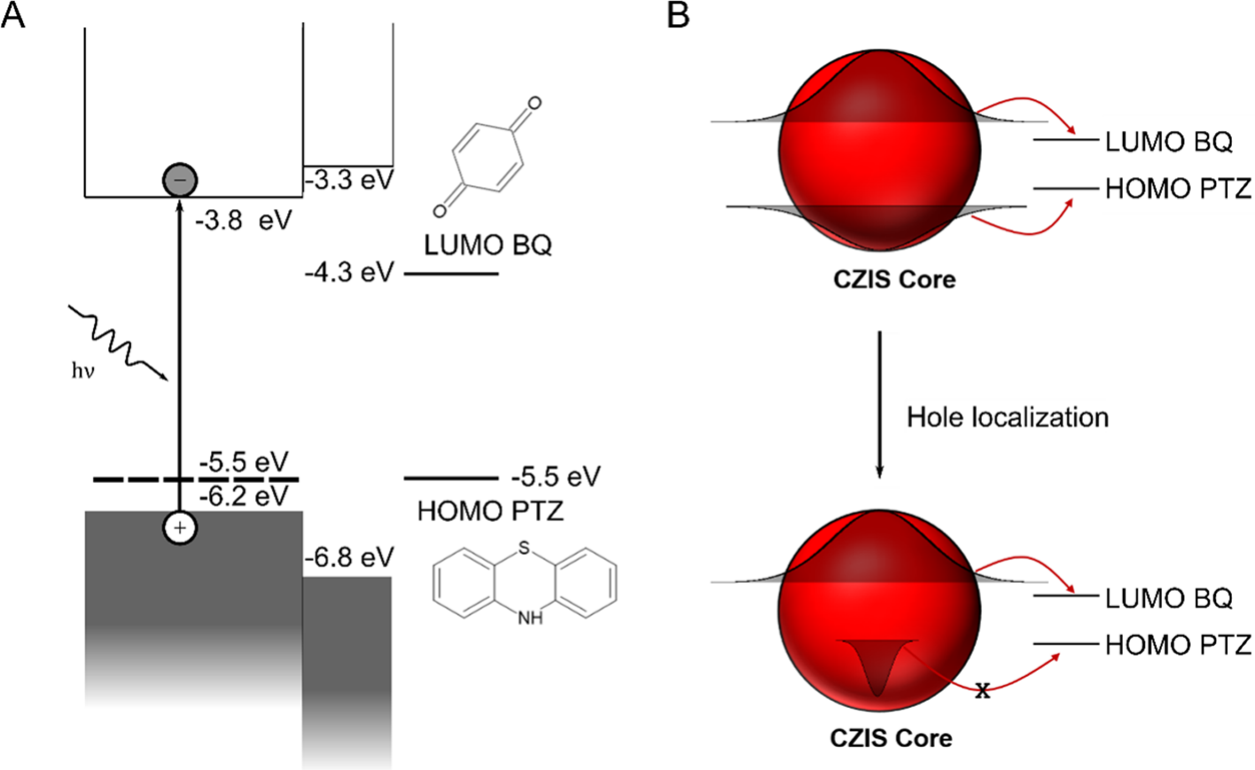
(A) Approximate Band Alignment between a 2.4 eV Bandgap CuInS_2_ Core,^55^ the ZnS Shell,^57^ and the Molecular Acceptors,^58^ According to the Literature. The Hole Localization Defects (CHS) Lie at About −5.5 eV According to the Same Cyclic Voltammetry Study.^55^ (B) Representation of the Difference in Hole Transfer between Delocalized and Localized Holes

Therefore, the electrons in the QD can be easily transferred to the acceptor because of a high overlap integral involving the QD CB and BQ LUMO wave functions. On the other hand, due to a lower overlap integral of the CHS orbitals with the PTZ HOMO, hole transfer in CIS is efficient only before the hole localization process. In other words, while the hole is still delocalized in the VB. This is illustrated in Scheme 1B.

Furthermore, the energy levels of the CHS and the PTZ HOMO may be very similar, further limiting the driving force for charge transfer (Scheme 1A). Finally, if the ZnS shell is thick enough, it will efficiently limit hole transfer, as seen for Cu_0.2_(Zn)­InS_2_/ZnS, since it will act as a barrier for positive charge carriers.

The limitation of our data presented in Figure 4 is that TAS is not very sensitive to holes in this case, and our TRPL measurements, which are equally sensitive to both charge carriers, have a time resolution of a few nanoseconds. Nonetheless, a change in the TAS decay for Cu_0.3_(Zn)­InS_2_ with PTZ is observed. In addition, the TRPL charge transfer efficiency differs from that of the PL data for Cu_0.3_(Zn)­InS_2_ (Table 2). Indeed, as shown earlier, PL measurements yield a hole transfer of about 40% for Cu_0.3_(Zn)­InS_2_ and 3% for Cu_0.2_(Zn)­InS_2_/ZnS. This discrepancy can be explained assuming that most of the observed TRPL signal originates from the population of Cu_0.3_(Zn)­InS_2_ QDs that had undergone hole localization before hole transfer to PTZ could have occurred.

Meanwhile, the quenching of the PL signal corresponds to all the transferred holes. This is further evidenced by the wavelength-dependence of the quenching efficiency (Φ_q_) of the PL signals. As shown in Figure S11, the higher the excess energy, the higher the Φ_q_. Therefore, photogenerated holes with higher temperatures will have a higher probability of transferring to PTZ before cooling down and localizing into the CHS. In line with the above, the emission spectrum with PTZ shows a small red shift, which can be explained by the effect of CHS states closer to the VB being more susceptible to hole transfer to PTZ, as it would be energetically more favorable (Figure S12).

In conclusion, the localized holes have a lower charge transfer efficiency, which is obtained from the TRPL lifetime quenching, and only the thin ZnS shell in Cu_0.3_(Zn)­InS_2_ permits hole transfer before CHS formation. The small quenching in Cu_0.2_(Zn)­InS_2_/ZnS corresponds to a very slow, low probability, hole transfer captured by TRPL and static PL in a similar way, with no ultrafast component. This is because the shell is too thick for the hole wave function to leak to the surface before CHS formation. Furthermore, according to our previous study,^26^ the samples without any Zn doping, Cu_0.3_InS_2_ and CuIn_0.4_S_2_, have a hole-trapping process unrelated to Cu, which is responsible for their significantly lower PLQY. A similar process would be occurring in Cu­(Zn)­In_0.8_S_2_, due to the lack of a detectable PL signal and the absence of a ZnS shell.

Consequently, it is impossible to observe the PTZ quenching in Cu­(Zn)­In_0.8_S_2_ due to the competition with ligand or surface state hole and electron trapping processes, affecting a large proportion of the photogenerated charge carriers.

The values for band edges and acceptors are taken from literature data of similar QDs,^55,58^ and are subject to changes due to the exact size of the core and the shell.^57–60^ For example, smaller bandgap QDs may increase the VB limit (up to *ca.* −5.1 eV for bulk). Alternatively, electrons may easily tunnel through the few-nanometer-thick shell because of their smaller effective mass.^61^ However, our observations allow us to conclude that the synthesis of Cu_0.2_(Zn)­InS_2_/ZnS produces a sufficiently thick ZnS shell to (i) efficiently passivate against hole trapping, (ii) extend the lifetime for recombination, while (iii) still allow the flow of electrons for charge transfer processes outside the QD, while only a few holes can be transferred over long time scales. Alternatively, the Cu_0.3_(Zn)­InS_2_ synthesis, without thermal post-treatment, provides a sufficient passivation to allow for much more efficient hole transfer, both fast and slow (free and localized holes). Another explanation for the reduced hole transfer compared to the electrons is the formation of a type II band alignment.^59^ Furthermore, we learned from these results that electron trapping is not a limiting process in the recombination of Cu-deficient CZIS, as electrons are not efficiently passivated from transfer toward the surface. The same cannot be said about the Cu-rich samples, since we have observed a considerable increase in the lattice disorder as corroborated by the Cu K-edge XAS spectra, which would translate into a larger number of internal trap states.^26^

## Conclusions

We have carried out a systematic study of CIS QDs with different levels of Zn doping and stoichiometry, combining optical and X-ray spectroscopies. We have observed that Zn can be incorporated both inside and around the core of the nanoparticle, forming a shell of ZnS. In Cu-deficient samples, Zn^2+^ ions form a ZnS shell when added during the synthesis without the application of any post-treatment of approximately half the volume compared to the shell growth treatment. The S K-edge could be a powerful probe for in situ monitoring of shell growth in colloidal dispersions of QDs. Furthermore, Zn K-edge measurements present signals compatible with an interstitial defect in Cu-deficient samples, although it does not have detrimental effects on the photophysics of the QDs.

In Cu-rich samples, Zn^2+^ is incorporated mainly as a substituent of Cu^+^ or In^3+^. We have shown the strength of XAS at the Zn and S K-edges to determine Zn incorporation and ZnS formation, which is not easily observable with TEM due to the similarity with the core. Furthermore, we presented how wavelet transforms of EXAFS data can help to identify interstitial defects.

From quenching studies with electron and hole scavengers, we have determined the formation of a ZnS shell, which prevents hole trapping at the surface, but it can be tailored to allow its transfer to an acceptor. On the other hand, electrons can easily be transferred through the barrier to an appropriate acceptor. The difference in transfer efficiency can be attributed to the localization of holes in the core and is, thus, compatible with the existence of CHS close to the VB edge.

## Supplementary Material



## Data Availability

The data that support the findings in this study are openly available at the IMDEA Nanoscience repository ref 62.

## Notes

Quantum dot characterization with XRD, HR-TEM, and XRF; bandgap estimation with TAS; complementary PL and UV–vis spectra; Cu K-edge XAS; FEFF fitting results; linear combination fitting of XANES spectra; shell thickness estimation. Wavelet transform analysis; TRPL analysis, and complementary PL spectra with quenchers (PDF)
