# Supplementary material for: Unveiling Zn Incorporation in CuInS2 Quantum Dots: X‑ray and Optical Analysis of Doping Effects, Structural Modifications, and Surface Passivation
Source: Chem Mater. 2026 Mar 9;38(6):2601–12. doi: 10.1021/acs.chemmater.5c01878 (PMC13020008; doi:10.1021/acs.chemmater.5c01878)
Supplement: Supplementary file 1 [file cm5c01878_si_001.pdf]

Supplementary Information

Unveiling Zn incorporation in $CuInS_2$ quantum dots: X-ray and optical analysis of doping effects, structural modifications and surface passivation

Andrés Burgos-Caminal[1,2,3,4*], Brener R. C. Vale[5,6], André F. V. Fonseca[8,6], Juan F. Hidalgo[1], Elisa P. P. Collet[1], Lázaro García,[2] Víctor Vega-Mayoral[1], Saül Garcia-Orrit[1], Iciar Arnay[1], Juan Cabanillas-González[1], Laura Simonelli[7], Ana Flávia Nogueira[8], Marco Antônio Schiavon[6], Thomas J. Penfold[9], Lazaro A. Padilha[5] and Wojciech Gawelda[2,1,10*]

1. Madrid Institute for Advanced Studies IMDEA Nanoscience, Ciudad Universitaria de Cantoblanco, Calle Faraday 9, 28049 Madrid, Spain
2. Departamento de Química, Universidad Autónoma de Madrid, Ciudad Universitaria de Cantoblanco, Calle Francisco Tomás y Valiente 7, 28049 Madrid, Spain
3. Departamento de Química Analítica, Química Física e Ingeniería Química, Universidad de Alcalá, Alcalá de Henares, Madrid 28805, Spain
4. Departamento de Química Física Aplicada, Universidad Autónoma de Madrid, Ciudad Universitaria de Cantoblanco, Calle Francisco Tomás y Valiente 7, 28049 Madrid, Spain
5. Instituto de Física Gleb Wataghin, Universidade Estadual de Campinas- UNICAMP, Campinas 13083-852 São Paulo, Brazil
6. Grupo de Pesquisa Química de Materiais, Departamento de Ciências Naturais, Universidade Federal de São João Del-Rei, Brazil
7. CELLS-ALBA Synchrotron Light Source, 08290 Cerdanyola del Vallès, Barcelona, Spain
8. Laboratório de Nanotecnologia e Energia Solar, Chemistry Institute, University of Campinas – UNICAMP, Campinas, São Paulo, Brazil
9. Chemistry, School of Natural and Environmental Sciences Newcastle University, NE1 7RU Newcastle upon Tyne, UK
10. Faculty of Physics, Adam Mickiewicz University, ul. Uniwersytetu Poznańskiego 2, 61-614 Poznań, Poland

**Corresponding author(s): andres.burgos@uam.es ; wojciech.gawelda@uam.es*

1. Quantum dot characterization

We characterized the physico-chemical properties of as-synthesized samples with three different techniques:

A) X-ray diffraction (XRD) of the samples deposited on a glass slide. We used a commercial Rigaku SmartLab SE multipurpose X-ray diffractometer. We employed a Bragg-Bentano geometry with Cu Kα (1.54 Å) as the source.

B) High-resolution transmission electron microscopy (HR-TEM) using a JEM-2100 from JEOL (Fig. S1).

C) Elemental analysis through X-ray fluorescence (XRF). We used an S2 PICOFOX from Bruker, obtaining the relative mass for each element.

Table S1. Elemental analysis of the stoichiometry through X-ray fluorescence. The result and its standard error is obtained from the average of three measurements.

	**Cu:In**	**Zn:Cu**	**Name**
A	2.41 ± 0.03		$CuIn_{0.4}S_2$
B	0.28 ± 0.03		$Cu_{0.3}InS_2$
C	1.25 ± 0.05	0.216 ± 0.001	$Cu(Zn)In_{0.8}S_2$
D	0.299 ± 0.009	2.810 ± 0.006	$Cu_{0.3}(Zn)InS_2$
E	0.245 ± 0.005	10.41 ± 0.01	$Cu_{0.2}(Zn)InS_2/ZnS$

The characterization results for $CuIn_{0.4}S_2$, $Cu_{0.3}InS_2$ and $Cu_{0.2}(Zn)InS_2/ZnS$ were previously published.[1]

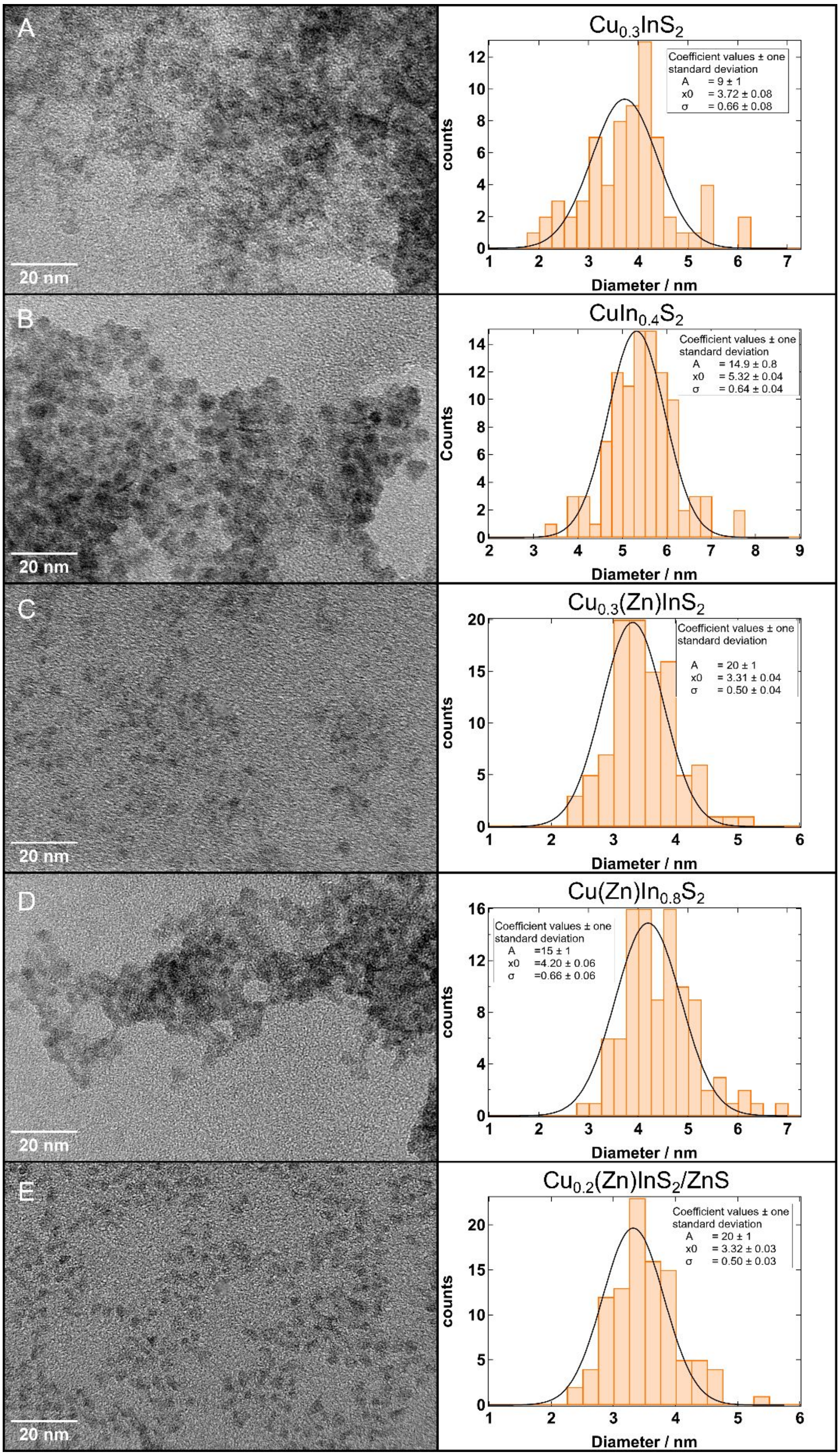

Figure S1. HR-TEM micrograph (left), and corresponding histograms (right) for the five samples under study. The histograms have been obtained with these and supplementary micrographs.

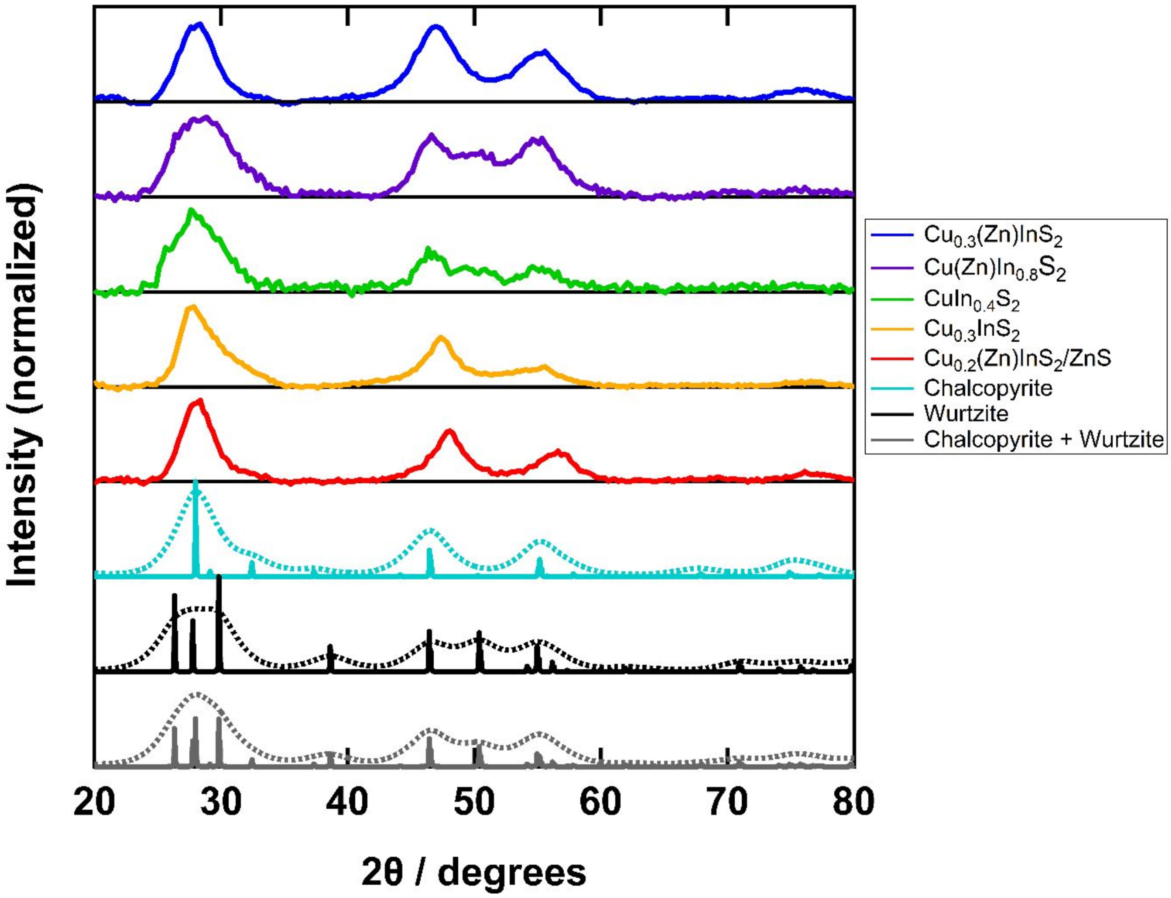

Figure S2. XRD patterns from the five samples and two reference spectra, chalcopyrite[2] and wurtzite,[3] calculated with VESTA,[4] and their average to simulate a mixture. The calculated spectra have been broadened through the convolution of the spectra with a pseudo-Voigt profile.

3. Bandgap estimation

The bandgap is estimated as the center of the ground state bleach (GSB) in a transient absorption measurement before trapping at midgap states (0.5 ps).

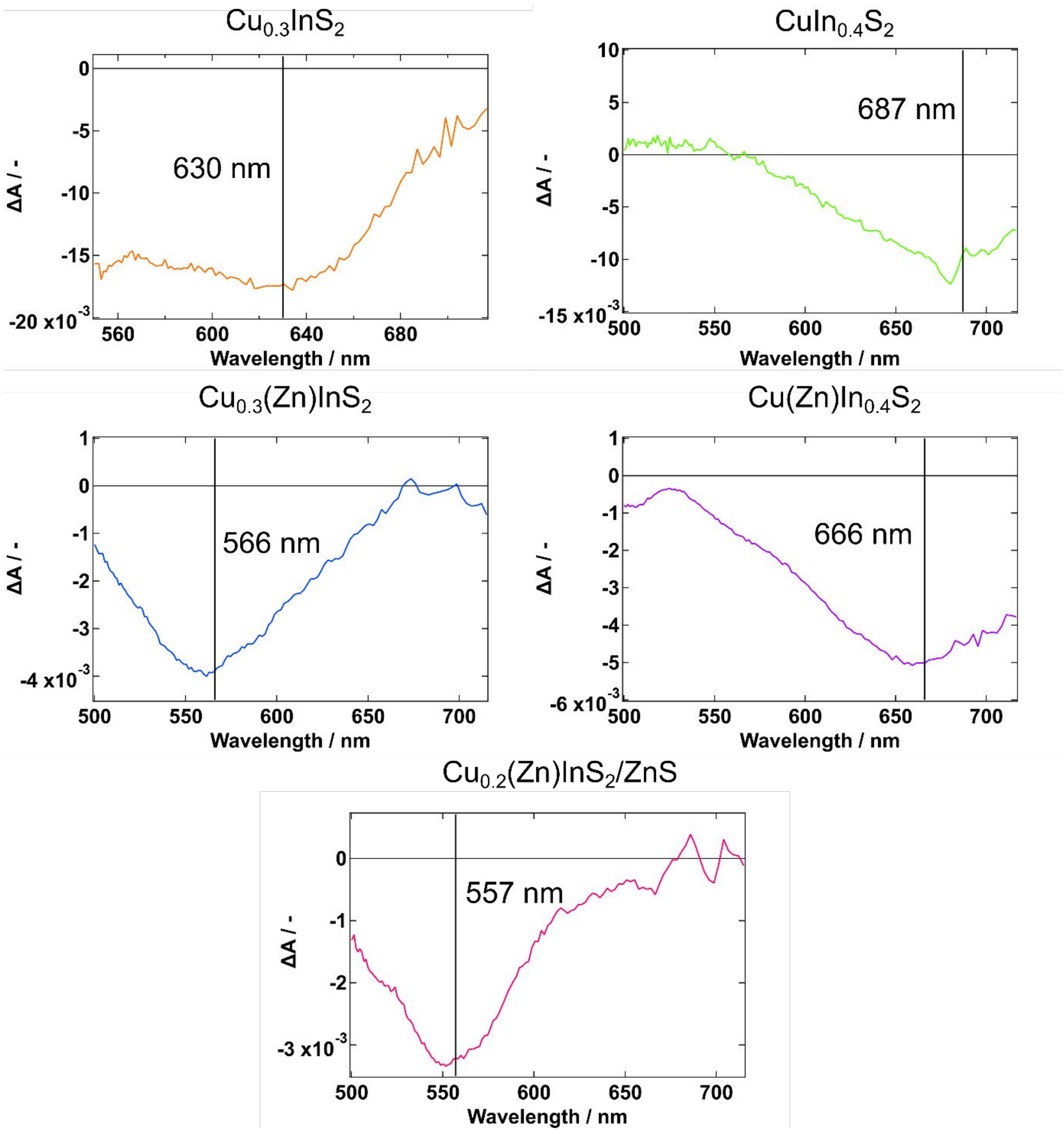

Figure S3. Transient absorption at 0.5 ps for each quantum dot sample. The center of the GSB is taken as the bandgap.

2. Complementary steady-state photoluminescence and UV-Vis absorption spectra

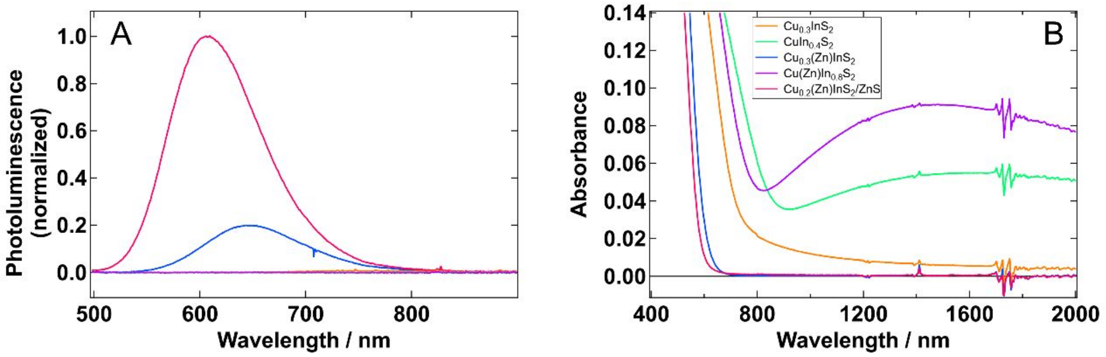

Figure S4: Linear photoluminescence spectra (A) and absorbance spectra (B) of the five samples extended to the near infrared, where the stoichiometric samples show what can be assigned to a Localized surface plasmon resonance (LSPR)

3. Cu K-edge XANES and EXAFS

Cu K-edge XAS measurements were carried out during the same beamtime as the Zn and S K-edge experiments shown in Fig. 2. The results for $Cu_{0.3}InS_2$, $CuIn_{0.4}S_2$ and $Cu_{0.2}(Zn)InS_2/ZnS$ were already published in our previous article.[5]

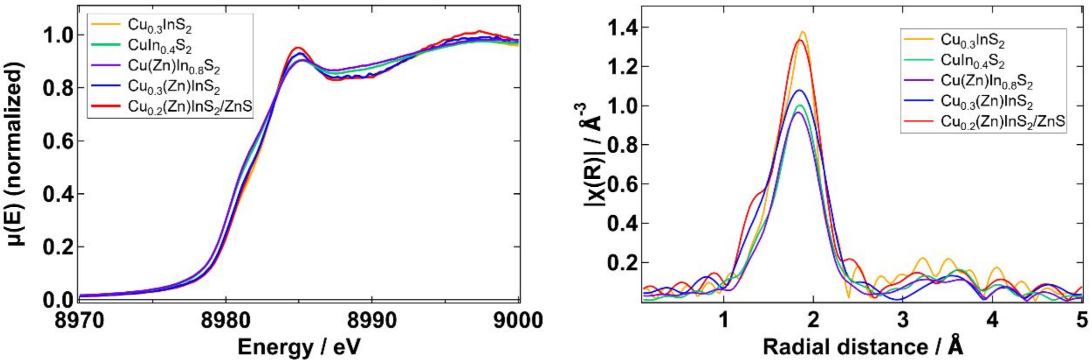

Figure S5: XANES (left) and EXAFS (right) spectra at the Cu K-edge for the five samples under study. Note the broader 1*s*→4*p* transition at 8984 eV for the stoichiometric samples, characteristic of a more disordered structure.

3.1 FEFF fitting results of Zn K-edge EXAFS

EXAFS analysis was carried out with the Artemis program,[6] applying the in-built FEFF6 for the fitting procedures. All samples were fitted in identical spectral ranges within the following limits:

k-range = 2.1 – 13 $Å^{-1}$
k-weight = 1
R-range = 1.25 – 3 Å

The background removal was carried out with k^2 weighting, a R_{bkg} of 1.2 Å and values of E_0 of 9661.2 and 9661.8 eV.

The crystal structure of ZnS was obtained from a reference and used for the FEFF calculation.[7] The resulting single scattering paths involving S atoms (1st coordination shell) were used to fit the experimental data according to EXAFS equation:[8]

$$\chi(k) = \sum_j \frac{S_0^2 N_j f_j(k) e^{-2R_j/\lambda(k)} e^{-2k^2\sigma_j^2}}{kR_j^2} \sin\left[2kR_j + \delta_j(k)\right], \qquad (S1)$$

where our aim was to obtain statistically reliable/meaningful fitting of N_j (coordination numbers), R_j, (first shell near neighbor distances,) and σ_j (mean square disorder or Debye-Waller factors) parameters. The values of S_0^2 can be obtained from the literature or from the fit of a well-known reference.

In our case we set the value of $S_0^2 = 0.7$ as a reasonable estimation obtained from an EXAFS fit of bulk ZnS reference sample data (see Table 1). A k weight of 1 was used for the fit in order to maximize the first shell S scattering signal. The best-fit results are summarized in Table S2 and depicted in Fig. S2.

Table S2: Zn K-edge EXAFS FEFF fitting parameters for the single scattering path with the first shell of S atoms to a Zincblende ZnS structure. The k1 results are used in the main text due to the smaller error.

Structure and fit	Sample	$N \cdot S_0^2$	N	σ^2	R	R-factor
**ZnS Zincblende k-weight = 1 fit**	$Cu(Zn)In_{0.8}S_2$	3.0 ± 0.4	4.3 ± 0.6	0.007 ± 0.002	2.30 ± 0.02	0.02411
	$Cu_{0.3}(Zn)InS_2$	2.5 ± 0.3	3.5 ± 0.5	0.005 ± 0.002	2.33 ± 0.01	0.02403
	$Cu_{0.2}(Zn)InS_2/ZnS$	1.9 ± 0.3	2.6 ± 0.4	0.004 ± 0.003	2.34 ± 0.02	0.03760
**ZnS Zincblende k-weight = 2 fit**	$Cu(Zn)In_{0.8}S_2$	3.7 ± 0.6	5.2 ± 0.9	0.010 ± 0.002	2.326 ± 0.007	0.03466
	$Cu_{0.3}(Zn)InS_2$	2.8 ± 0.5	3.9 ± 0.7	0.006 ± 0.002	2.338 ± 0.006	0.04041
	$Cu_{0.2}(Zn)InS_2/ZnS$	2.3 ± 0.5	3.2 ± 0.7	0.007 ± 0.003	2.343 ± 0.009	0.06051

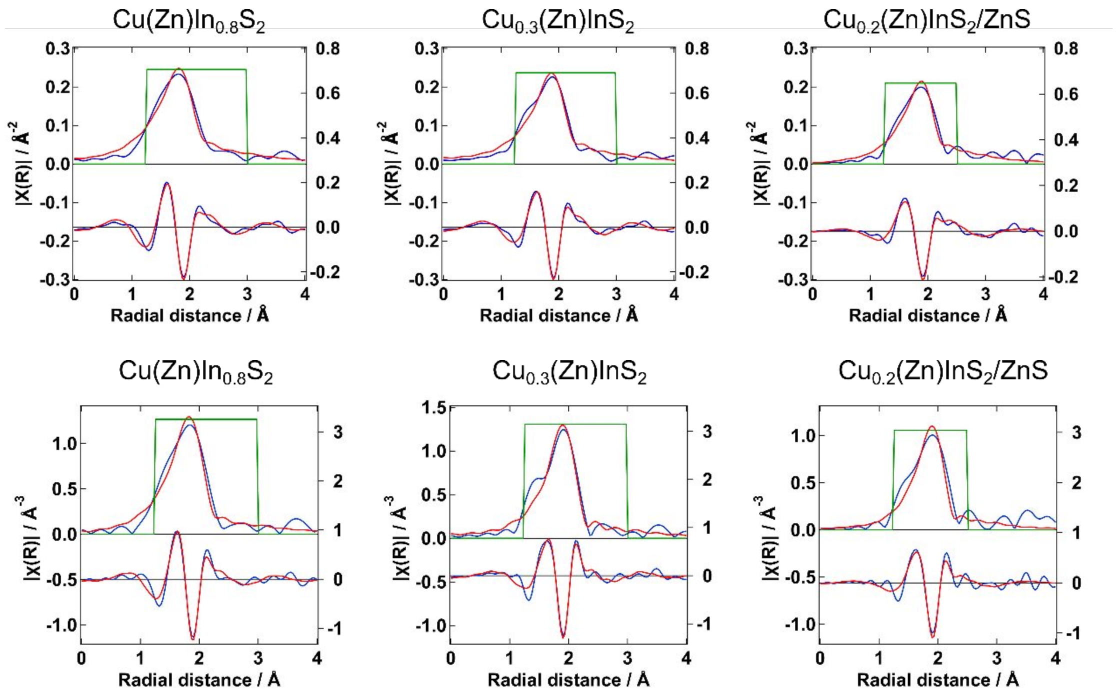

Figure S6: FEFF6 fits (red) for all CZIS QD samples (blue) at the Zn K-edge. The fits are carried out for a Zincblende ZnS structure. The actual disorder and mixture of structures in which Zn is located prevents us from obtaining excellent fits. However, it allows us a rough comparison between samples. The top plots correspond to a k-weight of 1 while the bottom ones correspond to one of 2.

3.2 Linear combination fitting of XANES spectra

The fitting of XANES spectra using a linear superposition of different QD composition data were performed with the Athena program of the Demeter package.[6] In Fig. S3 we show the results of such fitting using a linear combination of $Cu_{0.2}(Zn)InS_2/ZnS$ and $CuIn_{0.4}S_2$ spectra.

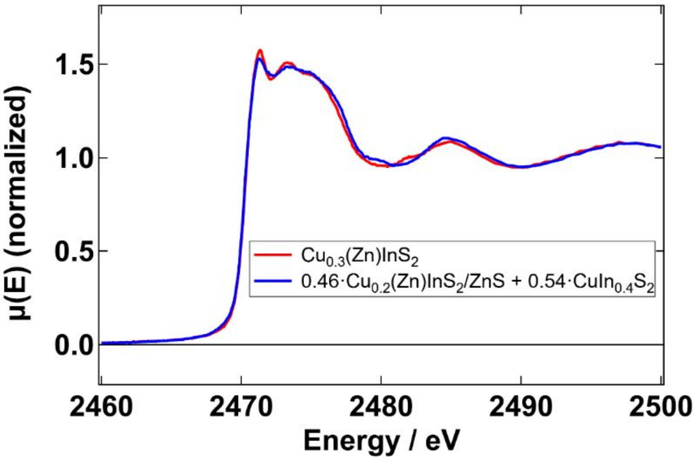

Figure S7: Linear combination fitting of $Cu_{0.3}(Zn)InS_2$ with $Cu_{0.2}(Zn)InS_2/ZnS$ and $CuIn_{0.4}S_2$.

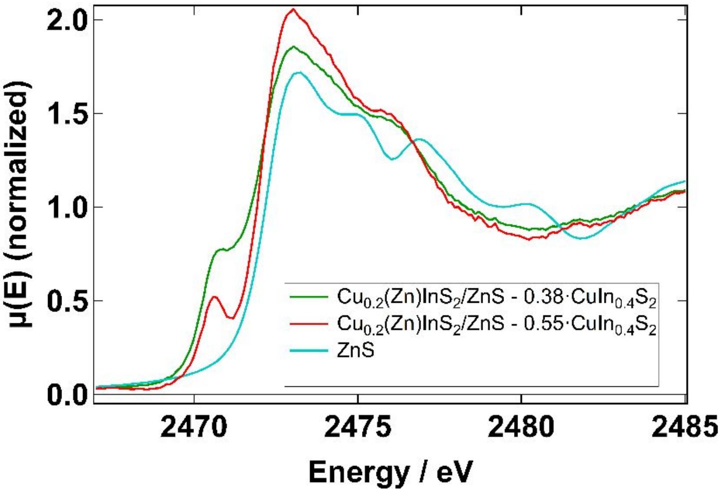

Figure S8: Extraction of the ZnS shell Sulfur K-edge spectrum. This is done subtracting the spectrum of $CuIn_{0.4}S_2$, which does not contain Zn, to that of Cu0.2(Zn)InS2/ZnS. Two examples are shown, by subtracting either 0.38 or 0.55 times the CuIn0.4S2 spectrum. One is closer to the ZnS spectrum around the 2474 eV area peak while the other agrees better in the 2471 eV area. Due to the slight differences between samples, it is not possible to completely remove the 2471 eV peak. In addition, the bulk ZnS spectrum shows additional oscillations. We propose that they originate from either multiple-scattering or second shell scattering that is not significant in the thin and disordered ZnS shell of the quantum dots.

3.3 Shell thickness estimation

If we consider the crystalline structures of Fig. 1.B, we can make an estimation of the shell thickness for $Cu_{0.2}(Zn)InS_2/ZnS$ and $Cu_{0.3}(Zn)InS_2$.

Let us assume spherical QDs. The radius (r) of the QD is given by the TEM measurement.

Considering $V = \frac{4}{3}\pi r^3$, we can obtain the Volume (V) from the radius or vice versa.

From to the crystal structures of Chalchopyrite[2] ($CuInS_2$) and zincblende[7] (ZnS) we can obtain the unit cell volume and the volume per S atom, obtaining 42.08 and 39.68 Å^3/S atom, respectively. Since both values are very close, we will consider them equal and directly translate proportion of S in each environment to volume.

With the proportion of S on the core and the shell we can estimate the radius of the core and the thickness of the shell (d). Based on Figs. S6 and S7 we consider proportions of 60% and 30% of ZnS for $Cu_{0.2}(Zn)InS_2$/ZnS and $Cu_{0.3}(Zn)InS_2$. With this, we calculate the volume of the core, its radius and the shell thickness.

Table S3: Shell thickness calculations based on the particle size obtained with TEM and the S proportions obtained from XAS.

QD	R_{tot} / nm	V / nm^3	V_{core} / nm^3	r_{core} / nm	d / nm
**$Cu_{0.2}(Zn)InS_2$/ZnS**	1.66	19.1	7.66	1.22	0.44
**$Cu_{0.3}(Zn)InS_2$**	1.66	19.0	13.3	1.47	0.19

For $Cu_{0.2}(Zn)InS_2$/ZnS we obtain a shell thickness of 0.44 nm, close to the unit cell length of 0.54 nm, explaining the passivation. Meanwhile $Cu_{0.3}(Zn)InS_2$ has a shell thickness of 0.19 nm, considerably smaller than the ZnS unit cell. This would correspond to a monolayer of Zn atoms on the surface, giving higher chances of charge transfer through the shell.

3.4 Wavelet transform analysis

Wavelet transform (WT) analysis was carried out using the XWave code,[9] employing a Morlet wavelet. The parameters used were:

Resolution = 0.01

η = 7.5

σ = 0.5

k-weight = 3

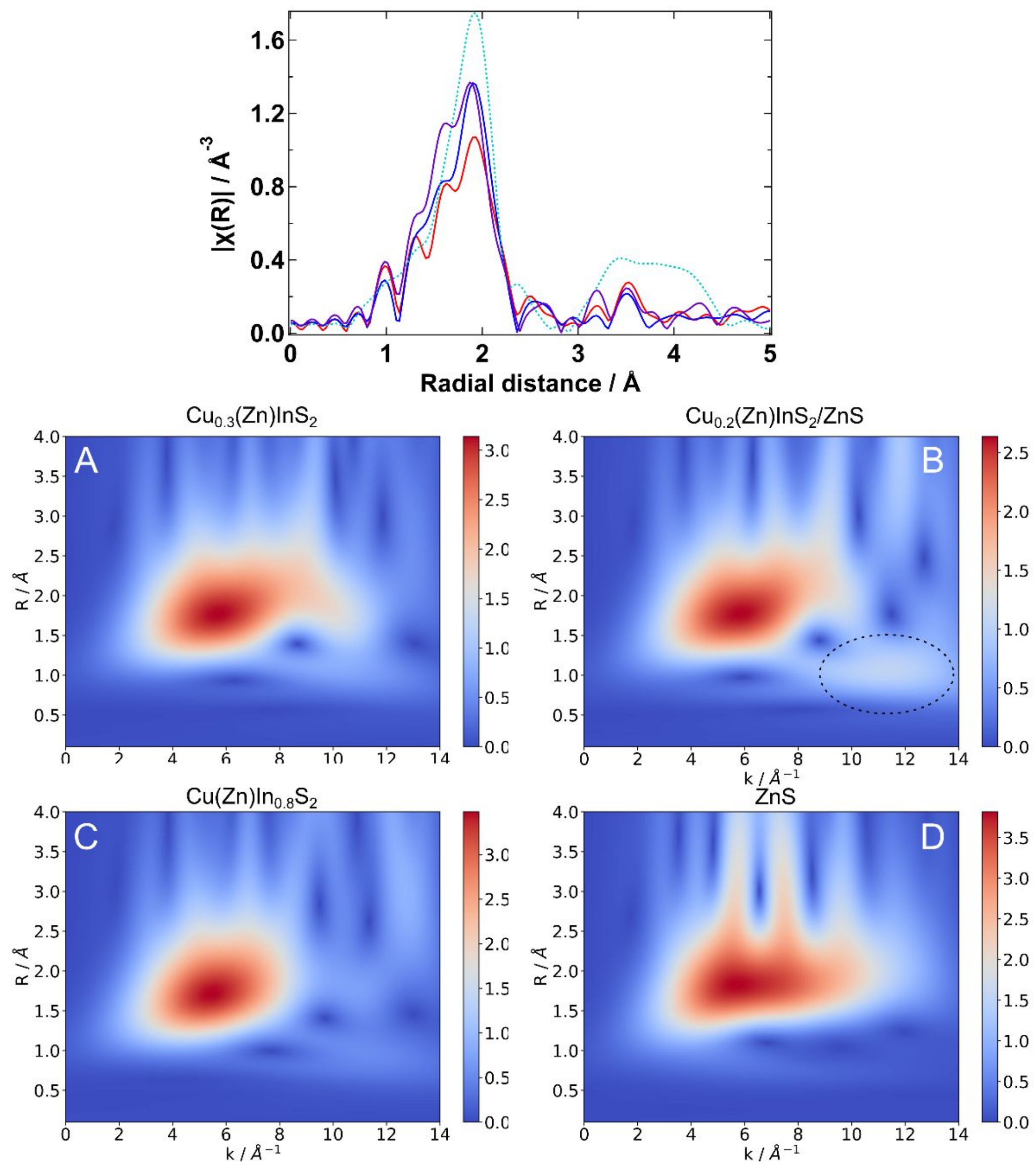

Figure S9: Alternative data treatment for the Zn K-edge EXAFS and wavelet calculation. The plots shown in Figs. 2 and 3 have been obtained applying a Rbkg value in Athena of 1.2, while these are obtained with a value of 0.95. This avoids suppressing the signal at around 1 Å, but introduces an artifact in the form of an oscillation across the spectrum.

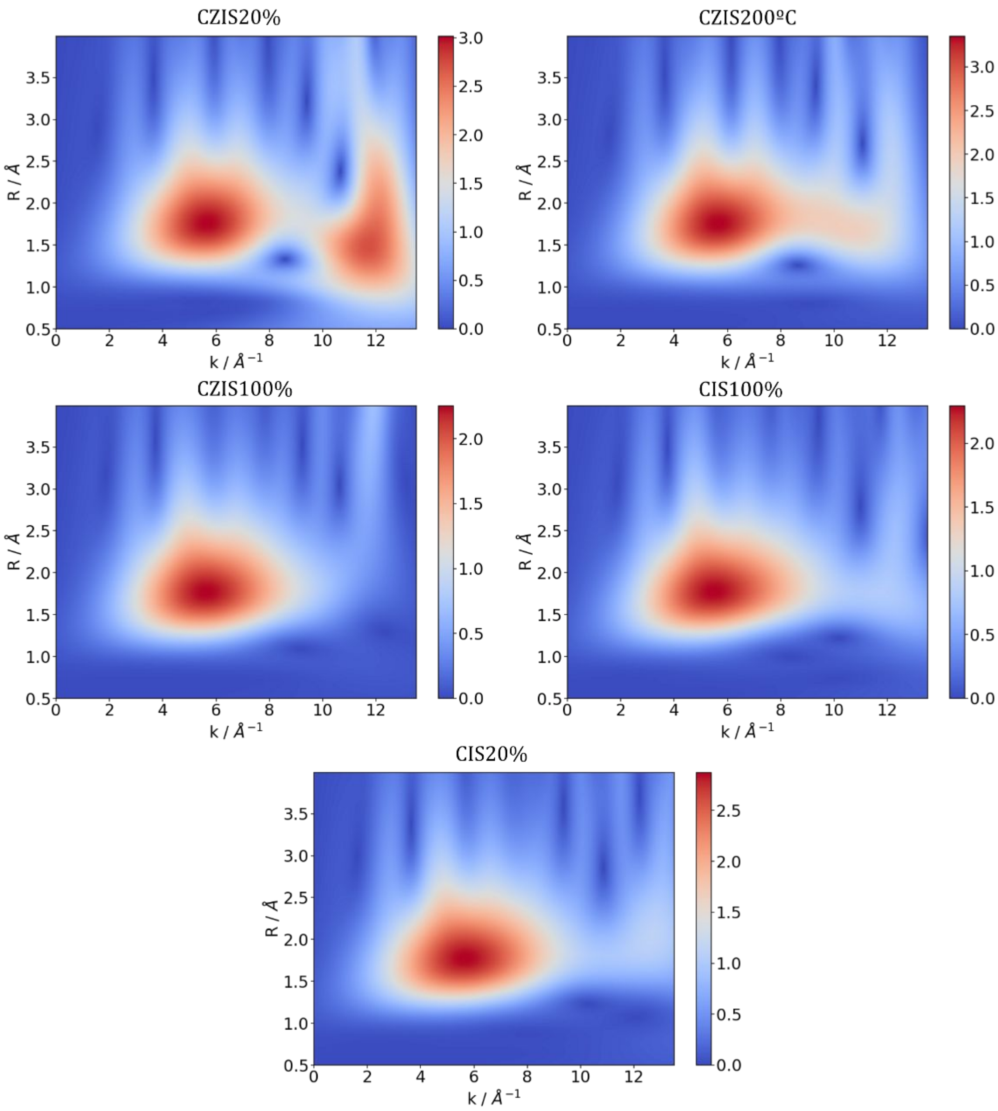

Figure S10: Wavelet transform of the Cu K-edge EXAFS of $Cu_{0.3}$(Zn)InS_2, $Cu_{0.2}$(Zn)InS_2/ZnS, Cu(Zn)$In_{0.8}S_2$, $CuIn_{0.4}S_2$, and $Cu_{0.3}InS_2$. We employed cubic k-weighting to maximize the contrast in the high k region of the spectra, the same as in case of Zn K-edge (Fig. 3 of the main text).

4. Time-resolved photoluminescence analysis

The time-resolved photoluminescence (TRPL) can also be fitted to a sum of exponential decays convoluted with Gaussian functions:

$$S(t) = A\frac{1}{2}\exp\left(\frac{4ln(2)w^2}{\tau^2} - \frac{t-t_0}{\tau}\right)\left(1 + \text{erf}\left(\frac{t-t_0}{4\sqrt{ln(2)}\,w} - \frac{2\sqrt{ln(2)}w}{\tau}\right)\right), \qquad (S2)$$

where w is the full with at half maximum (FWHM), t_0 is the time zero, A is the amplitude and τ is the lifetime.

From the fitting of TRPL data we obtain intensity-weighted averages of the lifetimes from a penta-exponential decay fit and compare them to obtain the quenching efficiency (Φ_q).

The results (Table S2) indicate lifetime quenching via hole transfer process of less than 10% for the most passivated sample ($Cu_{0.2}(Zn)InS_2/ZnS$), while it goes up to about 20% for $Cu_{0.3}(Zn)InS_2$. However, the multiexponential nature of the measured decays in these samples contributes to a larger uncertainty, compared to other systems, explaining the discrepancy with the time-integrated method showed in the main text. Nonetheless, comparing the decay traces in Fig. 4, we clearly observe that $Cu_{0.3}(Zn)InS_2$ is the most affected one, with the effect becoming present already at ultrafast timescales.

Table S4. Quenching results from intensity-averaged multiexponential fits. The standard errors are obtained from the fit errors and calculated with their propagation through the weighted average.

QD		QD-PTZ		QD-BQ	
TRPL	τ_{avg} / ps	τ_{avg} / ps	Φ_q	τ_{avg} / ps	Φ_q
$Cu_{0.2}(Zn)InS_2/ZnS$	288 ± 52	260 ± 160	0.09	14.3 ± 0.8	0.957
$Cu_{0.3}(Zn)InS_2$	220 ± 20	174 ± 15	0.217		

5. Complementary PL spectra

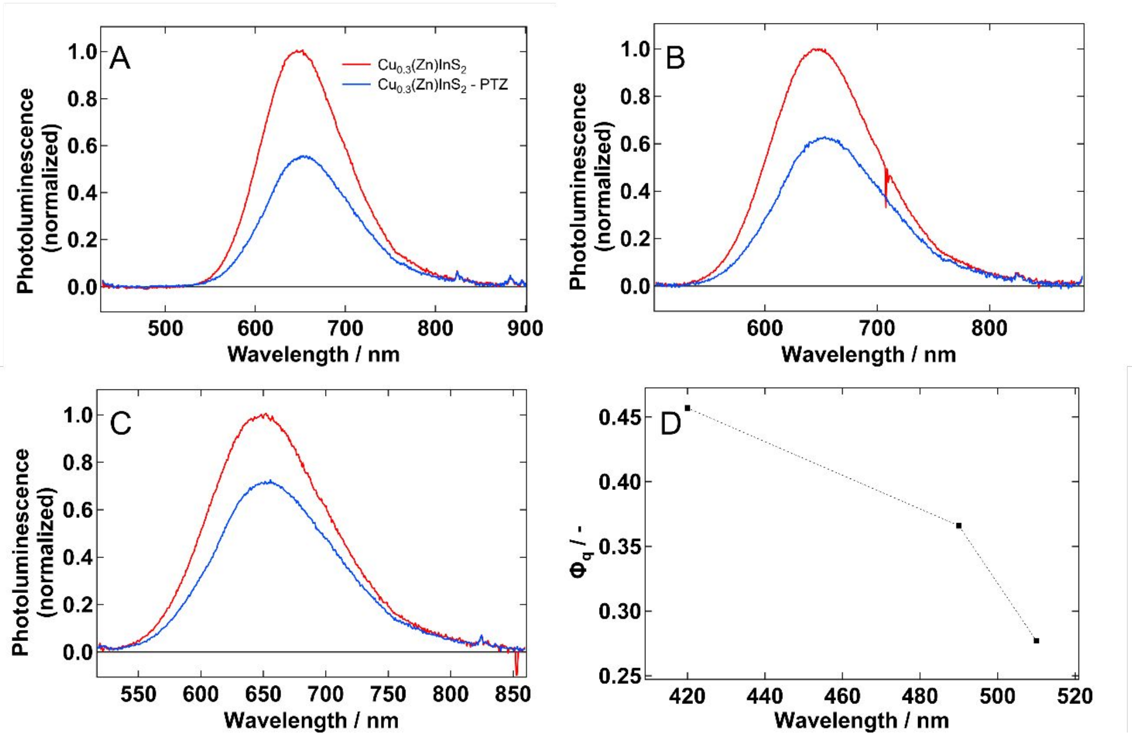

Figure S11: PL measurements of the quenching effects of PTZ where the excitation wavelength is A) 420 nm, B) 490 nm, and C) 510 nm. D) Wavelength-dependent quenching efficiency (Φ_q).

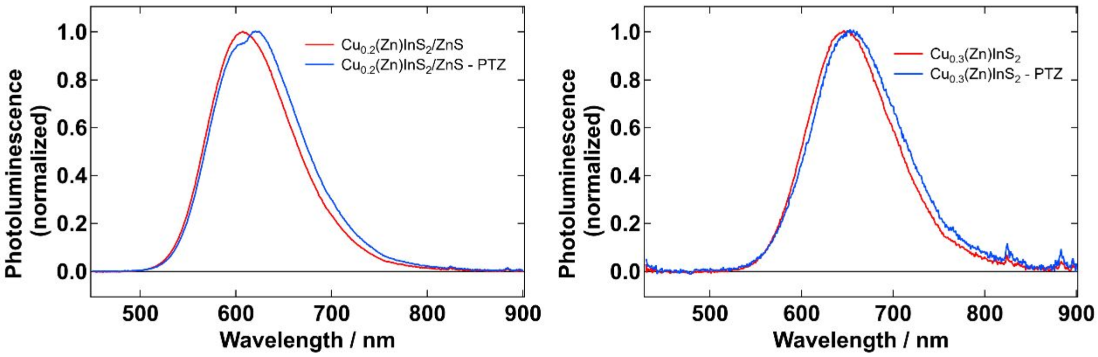

Figure S12: Normalized plots of $Cu_{0.2}(Zn)InS_2/ZnS$ (left) and $Cu_{0.3}(Zn)InS_2$ (right) with and without PTZ. The incorporation of PTZ slightly shifts the emission spectrum. CHS states located closer to the valence band will be more likely to finally transfer to PTZ, explaining the shift in the spectrum.

References

(1) Burgos-Caminal, A.; C. Vale, B. R.; V. Fonseca, A. F.; Collet, E. P. P.; Hidalgo, J. F.; García, L.; Watson, L.; Borrell-Grueiro, O.; Corrales, M. E.; Choi, T.-K.; Katayama, T.; Fan, D.; Vega-Mayoral, V.; Garcia-Orrit, S.; Nozawa, S.; Penfold, T. J.; Cabanillas-González, J.; Adachi, S.-I.; Bañares, L.; Nogueira, A. F.; Padilha, L. A.; Schiavon, M. A.; Gawelda, W. Selective Tracking of Charge Carrier Dynamics in CuInS2 Quantum Dots. *ACS Nano* **2025**, *19* (24), 21950–21961. https://doi.org/10.1021/acsnano.4c18469.

(2) Hahn, H.; Frank, G.; Klingler, W.; Meyer, A.-D.; Störger, G. Untersuchungen über ternäre Chalkogenide. V. Über einige ternäre Chalkogenide mit Chalkopyritstruktur. *Z. Für Anorg. Allg. Chem.* **1953**, *271* (3–4), 153–170. https://doi.org/10.1002/zaac.19532710307.

(3) Li, Q.; Zhai, L.; Zou, C.; Huang, X.; Zhang, L.; Yang, Y.; Chen, X.; Huang, S. Wurtzite CuInS2 and CuInxGa1−xS2 Nanoribbons: Synthesis, Optical and Photoelectrical Properties. *Nanoscale* **2013**, *5* (4), 1638–1648. https://doi.org/10.1039/C2NR33173J.

(4) Momma, K.; Izumi, F. Vesta 3 for Three-Dimensional Visualization of Crystal, Volumetric and Morphology Data. *J. Appl. Crystallogr.* **2011**, *44* (6), 1272–1276. https://doi.org/10.1107/S0021889811038970.

(5) Burgos-Caminal, A.; Vale, B. R. C.; Fonseca, A. F. V.; Collet, E. P. P.; Hidalgo, J. F.; García, L.; Watson, L.; Borrell-Grueiro, O.; Corrales, M. E.; Choi, T.-K.; Katayama, T.; Fan, D.; Vega-Mayoral, V.; García-Orrit, S.; Nozawa, S.; Penfold, T. J.; Cabanillas-Gonzalez, J.; Adachi, S.-I.; Bañares, L.; Nogueira, A. F.; Padilha, L. A.; Schiavon, M. A.; Gawelda, W. Selective Tracking of Charge Carrier Dynamics in CuInS2 Quantum Dots. arXiv December 19, 2024. https://doi.org/10.48550/arXiv.2412.15418.

(6) Ravel, B.; Newville, M. ATHENA, ARTEMIS, HEPHAESTUS: Data Analysis for X-Ray Absorption Spectroscopy Using IFEFFIT. *J. Synchrotron Radiat.* **2005**, *12* (4), 537–541. https://doi.org/10.1107/S0909049505012719.

(7) Jumpertz, E. A. Electron-Density Distribution in Zinc Blende. *Z. Für Elektrochem. Angew. Phys. Chem.* **1955**, No. 59, 419–425.

(8) Newville, M. EXAFS Analysis with Feff, Larch, Artemis, 2018. https://millenia.cars.aps.anl.gov/videos/FundamentalsOfXAFS/UsingFeff.pdf.

(9) Penfold, T. J.; Tavernelli, I.; Milne, C. J.; Reinhard, M.; Nahhas, A. E.; Abela, R.; Rothlisberger, U.; Chergui, M. A Wavelet Analysis for the X-Ray Absorption Spectra of Molecules. *J. Chem. Phys.* **2013**, *138* (1), 014104. https://doi.org/10.1063/1.4772766.
